# Analysis of Nipah Virus Codon Usage and Adaptation to Hosts

**DOI:** 10.3389/fmicb.2019.00886

**Published:** 2019-05-08

**Authors:** Rekha Khandia, Shailja Singhal, Utsang Kumar, Afzal Ansari, Ruchi Tiwari, Kuldeep Dhama, Jayashankar Das, Ashok Munjal, Raj Kumar Singh

**Affiliations:** ^1^Department of Biochemistry and Genetics, Barkatullah University, Bhopal, India; ^2^Gujarat Biotechnology Research Centre (GBRC), Department of Science and Technology, Government of Gujarat, Gandhinagar, India; ^3^Department of Veterinary Microbiology and Immunology, College of Veterinary Uttar Pradesh Pandit Deen Dayal Upadhyaya Pashu Chikitsa Vigyan Vishwavidyalaya Evam Go Anusandhan Sansthan (DUVASU), Mathura, India; ^4^Division of Pathology, ICAR-Indian Veterinary Research Institute, Bareilly, India; ^5^ICAR-Indian Veterinary Research Institute, Bareilly, India

**Keywords:** Nipah virus, codon usage, CAI, RCDI, similarity index, selection pressure, compositional constraint

## Abstract

A recent outbreak of Nipah virus (NiV) in India has caused 17 deaths in people living in districts of Kerala state. Its zoonotic nature, as well as high rate of human-to-human transmission, has led researchers worldwide to work toward understanding the different aspects of the NiV. We performed a codon usage analysis, based on publicly available nucleotide sequences of NiV and its host adaptation, along with other members of the *Henipavirus* genus in ten hosts. NiV genome encodes nine open reading frames; and overall, no significant bias in codon usage was observed. Aromaticity of proteins had no impact on codon usage. An analysis of preferred codons used by NiV and the tRNA pool in human cells indicated that NiV prefers codons from a suboptimal anticodon tRNA pool. We observed that codon usage by NiV is mainly constrained by compositional and selection pressures, not by mutational forces. Parameters that define NiV and host relatedness in terms of codon usage were analyzed, with a codon adaptation index (CAI), relative codon deoptimization index (RCDI), and similarity index calculations; which indicated that, of all hosts analyzed, NiV was best adapted to African green monkeys. A comparative analysis based on the relative codon deoptimization index (RCDI) for host adaptation of NiV, Hendra virus (HeV), Cedar virus (CedV), and Hendra like Mojiang virus (MojV) revealed that except for dogs and ferrets, all evaluated hosts were more susceptible to HeV than NiV.

## Introduction

The Nipah virus (NiV) is an RNA virus in the *Henipavirus* genus, *Paramyxoviridae* family, that infects both wild animals and humans (Gurley et al., 2017). In 1998, NiV disease was reported for the first time in Malaysia and the mortality rate associated with it was very high (40%) (Wacharapluesadee et al., 2010). Human-to-human transmission was not observed in the Malaysian outbreak; however, it was observed during the outbreaks in Bangladesh and India with a high mortality rate of 70% (Hsu et al., 2004; Chong et al., 2008; Wacharapluesadee et al., 2010; Arankalle et al., 2011). Thereafter, the virus has been detected in several countries such as China (Li et al., 2008), Cambodia (Reynes et al., 2005), Malaysia (Mohd Nor et al., 2000), Vietnam (Hasebe et al., 2012), the Philippines (Ching et al., 2015), Indonesia (Sendow et al., 2010), Thailand (Wacharapluesadee et al., 2005), Ghana (Hayman et al., 2008), Madagascar (Iehlé et al., 2007),and Timor-Leste (Heymann, 2008); though in many countries, NiV detection has been limited to serum antibody detection only (Olson et al., 2002; Hayman et al., 2008; Hayman et al., 2011). NiV is a zoonotic pathogen transmitted by fruit bats, namely *Pteropus* spp. (Epstein et al., 2006b) such as *P. vampyrus*, *P. hypomelanus*, *P. lylei*, and *P. giganteus*. These bats have been associated with outbreaks of NiV (Wacharapluesadee et al., 2010; Hasebe et al., 2012; Yadav et al., 2012; de Wit and Munster, 2015). The virus is regarded as a biosafety level-4 pathogen, presenting a great threat to human health as well as security, because of the high rate of mortality associated with its infection, along with the lack of an efficacious treatment regimen and vaccine (Epstein et al., 2006a; Rahman and Chakraborty, 2012). NiV causes respiratory and neurological illness among humans. The clinical signs and symptoms of its infection include fever with labored breathing, cough, headache, encephalitis and seizures (Ang et al., 2018).

Recently, in May 2018, a NiV outbreak in India claimed 17 lives in Kerala. Experimentally, various species of mammals have been found susceptible to NIV infection, hence serosurveillance studies play a crucial role in understanding the viral epidemiology (Kulkarni et al., 2013; de Wit and Munster, 2015; Singh et al., 2019).

The RNA of NiV is single stranded, with six consecutive genes from 3′–5′ direction namely N (nucleocapsid), P (phosphoprotein), M (matrix), F (fusion), G (glycoprotein), and L (large polymerase), and possesses nine coding sequences (CDSs). Of these genes, two encode integral membrane proteins; one encodes a glycoprotein (G) that is essential for attachment, and another encodes a fusion (F) protein needed for cell entry. Both the G (602 aa residues) and F (546 aa residues) proteins of NiV are essential to mediate the cellular entry into the hosts (Bossart et al., 2005; Bose et al., 2014). The M protein (352 aa residues) facilitates virus budding and morphogenesis. The L (2245 aa residues), N (532 aa residues), and P protein (709 aa residues) together form an RNA-dependent RNA polymerase (RdRP). The P gene encodes four proteins (P, V, W, and C), which are 709, 52, 532, and 166 residues in length, respectively (Kulkarni et al., 2009). The C, V, and W proteins are not important for virus replication; rather, they impart virulence. The V and W proteins result from RNA editing, whereas the C protein is encoded in an alternative open reading frame (ORF) that starts 23 nucleotides downstream of the P ORF (Schomacker et al., 2004).

At present, there is no prophylaxis for NiV; however, vaccination and passive transfer of antibodies as interventions against NiV have been evaluated (Broder et al., 2013). Subunit vaccine candidates using G and F protein with adjuvants like Alhydrogel and CpG oligodeoxynucleotides have shown protection in several animal models (Pallister et al., 2013). Other viral vectored vaccines in development strategies imply usage of either G or N protein of NiV (Satterfield et al., 2016); however, so far, no such attenuated vaccine candidate has been developed that contains all nine coding sequences. NiV virus-like particles (NiV VLPs) composed of G, F, and M proteins have been found to get processed like virus particle and evoke neutralizing immune response in mice (Walpita et al., 2011) and hamster models (Walpita et al., 2017). Experimental research on NiV vaccine candidates as well as the identification of suitable experimental animal models are needed to limit the impact of NiV infections.

The purpose of the present study was to elucidate codon usage pattern of NiV, that can be applied to generate an attenuated NiV vaccine strain. An international coalition of governments and pharmaceutical companies was formed in 2017 to develop safe, effective as well as affordable vaccines to combat pandemic diseases like NiV (Satterfield, 2017). The present study on codon usage by NiV will pave the way to design strategies to modify the NiV genome to reduce virulence as well as reveal replicative efficacy of the virus in human hosts, for the development of a safe and effective vaccine. This can be done by elucidating the least preferred codons for use in a synthetic attenuated vaccine engineering (SAVE) approach. Several animal models have been developed that facilitate the understanding toward the NiV pathogenesis and will be helpful for vaccination studies (Broder et al., 2016). NiV is known to infect bats, humans, pigs, horses, dogs, and cats (Hooper et al., 2001; Mungall et al., 2006; Mills et al., 2009). Experimental animal models for NiV infection include hamsters, cats, pigs, ferrets, squirrel monkeys, and African green monkeys (Geisbert et al., 2010; Satterfield et al., 2015). Hence, these 10 hosts have been selected in the present study to evaluate the codon usage in NiV.

Systems biology is an interdisciplinary approach that systematically describes the complex interactions between biological processes and can efficiently predict the behavior of the biological system (Oberg et al., 2011). Using this approach, we attempted to elucidate the codons usage pattern of NiV and its hosts. In addition, various pressures such as mutational, selectional, and compositional pressures, which are responsible for shaping the pattern of codon usage by NiV, are discussed. This is the first report of its kind in which we have analyzed a total of 10 hosts for their expression of NiV proteins, adaptation of the virus, and effects of the host on NiV codon usage. The adaptation of the same 10 hosts for other virus members of the genus *Henipavirus* [Hendra virus (HeV), Cedar virus (CedV) and Hendra like Mojiang virus (MojV)] (Wu et al., 2014) has also been elucidated for comparative analysis. Such analysis of hosts may provide insight to potential reservoir hosts, susceptible species and excellent experimental models of NiV. The information obtained in the study may also be useful in the rational design of an attenuated NiV strain that may have vaccine potential and a broader applicability.

## Materials and Methods

### Data Collection

Sequences of all the nine CDSs (G, F, M, N, L, P, C, V, and W) for NiV were retrieved from the National Center for Biotechnology Information (NCBI)^1^ in FASTA format. For G, F, M, N, L, P, and C, complete sequences were obtained; whereas, for V and W, partial sequences were used in this study. Sequences downstream of the RNA editing site were excluded for V and W genes. A total of 149 CDSs corresponding to 101500 codons were analyzed in the present study. Similarly, 181 CDSs of HeV, 21 CDSs of CedV and 14 CDSs of MojV corresponding to 118113, 16413, and 10408 codons, respectively, were retrieved.

### Overall Nucleotide Content Analysis

The nucleotide composition of CDSs, specifically the nucleotide at the third codon position (U3%, G3%, C3%, and A3%) and the overall AU% (total A and U nucleotides available), AU3% (nucleotides A and U present at third position of codon), GC% (total G and C nucleotides present), GC12 (the average of nucleotide G and C present at first and second positions of codon), and GC3 (total G and C nucleotides present at third position) were analyzed.

### Relative Dinucleotide Abundance Analysis

Variation in the frequency of dinucleotide pairs may affect the codon usage. Dinucleotide frequency is often used to determine whether some dinucleotide pairs are favored by an organism or not. A maximum of 16 dinucleotide combinations are feasible. The patterns of dinucleotide frequency indicate both selectional and mutational pressures; which was calculated using the following formula:

$$P_{\text{xy}}=\frac{F_{\text{xy}}}{F_{\text{x}}F_{\text{y}}}$$

where *f*_x_ and *f*_y_ are the frequency of individual nucleotides (*x* and *y*, respectively), and *f*_xy_ is the frequency of dinucleotides (xy) in the same sequence.

The ratio of the observed to expected dinucleotide frequency is known as odds ratio; if this ratio is more than 1.25, the dinucleotide is considered overrepresented, whereas values below 0.78 indicate a underrepresentation (Kunec and Osterrieder, 2016).

### Relative Synonymous Codon Usage (RSCU) Analysis

As described by Sharp and Li (1986), the RSCU value is the ratio of the observed to the expected value for a given amino acid. The RSCU value remains unaffected by the length of a sequence or amino acid frequency. Overrepresented codons possess RSCU values of more than 1.6, whereas underrepresented codons have values less than 0.6. Codons with RSCU values ranging between 0.6 and 1.6 are considered unbiased or randomly used. RSCU values of all hosts except *P. vampyrus* were obtained from the codon usage database^2^, and NiV RSCU data was analyzed using the CAIcal server^3^ (Puigbo et al., 2008). To determine *P. vampyrus* codon usage, 261 ORFs, (corresponding to a total of 138,222 codons) of a *P. vampyrus* “Shadow” isolate from the Lubee Bat Conservancy (female organism, kidney tissue; shotgun sequence) were analyzed using the ‘Countcodon’ program of Yasukazu Nakamura^4^.

### Average Hydropathicity (GRAVY) and Aromaticity (AROMO)

The GRAVY value is the sum of hydropathy values of all amino acids in a sequence divided by the number of residues (Kyte and Doolittle, 1982), which range between −2.0 and +2.0. Positive values indicate hydrophobicity of a protein, whereas negative values indicate hydrophilicity.

The AROMO value is the frequency of aromatic amino acids, i.e., Phe, Tyr, and Trp, in a given amino acid sequence.

### Similarity Index Analysis

The similarity index indicates the effect of host codon usage on pathogen codon usage. Codon usage by different genes of NiV and by hosts was analyzed. This strategy was developed by Zhou et al. (2013) to determine the potential for a host to harbor a virus. Using this method, the similarity of codon usage by a host and pathogen was estimated by considering 59 codons to be 59 different spatial vectors. The similarity index was calculated according to the following formula:

$$R(A,\;B)=\frac{\sum_{i=1}^{59}\mathrm{ai}*\mathrm{bi}}{\sum_{i=1}^{59}a^{2}*\sum_{\text{i}=1}^{59}b^{2}}$$

$$D(A,\;B)=\frac{1-R(A,\;B)}{2}$$

where ‘ai’ is the RSCU value for a specific codon for the NiV coding sequence, ‘bi’ is the RSCU value for the similar codon of the host. *D*(*A*, *B*) is a numerical value, ranging between 0.0 and 1.0 representing the effect of the overall codon usage of the host on that of NiV. The higher the value, the greater the impact of a host is on NiV codon usage.

### Analysis of the Effective Number of Codons (Nc)

In a coding sequence, most amino acids (except Met and Trp), are encoded by two or more codons, known as synonymous codons. To identify the bias in the use of synonymous codons, Wright (1990) given the concept of the effective number of codons (Nc). Nc values range from 20 to 61. A value of 20 indicates extreme bias, and means, despite the availability of synonymous codons, the amino acid is encoded by one codon only. Whereas a value of 61 indicates no bias in the codon usage; and means that all the available codons are used equally. Generally, if the observed Nc value is less than 35, the genome is considered to have highly biased codon usage. An analysis of variance (ANOVA) was calculated to determine whether the Nc values for each gene of NiV were significantly different.

### Neutrality and Parity Analysis

A neutrality plot was used to analyze the influence of mutation bias and translation selection on codon usage. A regression line was plotted between GC12 and GC3 contents. A slope of the regression line is indicative of the mutational force. A parity rule 2 (PR2) bias was calculated based on the AT bias [A3/(A3 + T3)] as the ordinate and GC bias [G3/(G3 + C3)] as the abscissa (Chen et al., 2014; Wu et al., 2015).

### Codon Adaptation Index (CAI) Analysis

Codon adaptation index (CAI) is a quantitative value that indicates how frequently a favored codon is used amongst highly expressed genes. It is a primary indicator of translation efficiency and is used to engineer nucleotide sequences to obtain maximal protein production for vaccine purposes (Gustafsson et al., 2012). CAI values range between 0.0 and 1.0; with higher values indicating a higher gene expression potential. Further, values close to 1 indicate that codons with higher RSCU values are being used in the gene. The CAI value is sequence length independent; this value depends only on the amino acid frequency (Xia, 2007). In the present work, CAI values were calculated for all nine CDSs of NiV individually, using an RSCU reference set for each organism.

### Relative Codon Deoptimization Index (RCDI)

Mueller et al. (2006) developed the RCDI, which compares the similarities in codon usage by genes and reference genomes. RCDI values provide an estimate of the rate of viral gene translation in a host genome. If codon usage by a pathogen and host is similar, the RCDI value is close to one, and the higher translation rate can be predicted (Butt et al., 2016) as well as being indicative of a greater adaptation to the host. RCDI values of the other four viruses of the *Henipavirus* genus (NiV, HeV, CedV, and MojV) were also calculated for the selected 10 hosts.

### Software and Tools Used

The RSCU values were determined using CODONW 1.4.2^5^. The program was also used to obtain AROMO values. GRAVY values were calculated using http://www.gravy-calculator.de/. CAI and RCDI values were also calculated using http://genomes.urv.es/CAIcal/ (Puigbo et al., 2008). The tRNA database was retrieved using an online tool (GtRNAdb: Genomic tRNA database^6^).

## Results

### Nucleotide Composition Analysis

In the present study, we analyzed a total of 149 CDSs corresponding to 101,500 codons of all nine CDSs (G, F, M, N, L, P, C, V, and W) of various NiV isolates. The general sequence information of samples including the year of isolation, host and type of sample (tissue/swab/urine/cerebrospinal fluid) are depicted in the Supplementary Table S1.

### A/U Richness of the NiV Genome

The NiV CDSs were found to be rich in A and U nucleotides, in comparison to G and C nucleotides. To determine the compositional constraints in the NiV genome, the nucleotide compositions of the CDSs were determined (Supplementary Table S2). Mean usage (%) of A was the highest (32.69 ± 0.1.51%) amongst the four nucleotides; U (26.76 ± 2.67%) revealed the highest value after A, followed by G (22.9 ± 2.25%), while C revealed the lowest value (19.34 ± 0.97%). Similar trends in nucleotide composition mean values were observed in the third positions of synonymous codons (A3%, U3%, G3%, and C3%); A3% (30.46 ± 3.5) and U3% (29.38 ± 4.29) were higher than G3% (20.8 ± 5.71) and C3% (19.36 ± 2.03). The mean AU and GC compositions were 57.76 ± 2.84% and 42.24 ± 2.84%, and the mean AU3 and GC3 compositions were 59.84 ± 5.29% and 40.17 ± 5.29%, respectively (Supplementary Table S2). The AU3% ranged from 43.11 to 64.81, whereas the GC3% ranged between 35.2 and 59.6. Overall, the NiV CDSs were found to be rich in A and U nucleotides, in comparison to G and C nucleotides.

### Dinucleotide Frequencies and Relative Synonymous Codon Usage (RSCU) Analysis

In the present study, ApA (10.4%) was observed as the most abundant dinucleotide reflecting a high abundance of A in the NiV genome. Whereas the least abundant dinucleotide pair was CpG (1.8%) (Supplementary Table S3) with the lowest odds ratio (0.258).

In order to determine the patterns of synonymous codon usage, RSCU values were calculated. RSCU values for the individual gene, as well as for the complete genome, were analyzed separately. All overrepresented codons are highlighted in their respective genes (Table 1). The average RSCU of NiV was compared with the RSCU of its natural hosts and experimental model hosts (Table 2). If a particular codon has the highest RSCU value in virus as well as in a host, this is considered as evidence of a shared codon preference.

**Table 1:** Relative synonymous codon usage (RSCU) analysis of the various codons of all coding sequences (G, F, M, N, L, P, C, V, and W) belonging to six genes of Nipah virus (NiV) genome.

S. no.	Amino acid	Codons	Coding sequences (CDSs)								
			C	F	G	M	N	P	L	W	V
(1)	Phenylalanine (F)	UUU	0.50	**1.56**	0.94	0.91	**1.13**	**1.16**	**1.17**	**1.47**	**1.45**
		UUC	**1.50**	0.44	**1.06**	**1.09**	0.87	0.84	0.83	0.53	0.55
(2)	Leucine (L)	UUA	1.09	**1.34**	0.94	0.24	0.46	0.47	**1.46**	0.25	0.24
		UUG	**1.91**	1.20	1.13	1.19	0.96	1.17	1.25	1.13	1.06
		CUU	0.55	0.90	0.87	1.22	0.99	1.27	1.13	0.84	0.90
		CUC	0.55	0.73	0.21	**1.41**	**1.82**	0.44	0.54	0.49	0.49
		CUA	0.82	0.88	**1.37**	0.86	1.14	0.89	0.95	1.47	1.46
		CUG	1.09	0.97	1.48	1.09	0.63	**1.75**	0.67	**1.82**	**1.85**
(3)	Isoleucine (I)	AUU	0.63	**1.15**	0.90	**1.39**	0.86	**1.48**	0.90	**1.60**	**1.57**
		AUC	**1.97**	0.87	**1.10**	1.19	**1.29**	0.83	0.85	0.72	0.78
		AUA	0.39	0.97	1.00	0.42	0.86	0.70	**1.25**	0.68	0.66
(4)	Valine (V)	GUU	0.50	**1.62**	**1.47**	**1.78**	**1.43**	**1.70**	1.25	**1.71**	**1.63**
		GUC	0.50	0.92	0.64	0.64	0.95	0.77	0.69	0.49	0.55
		GUA	1.32	0.89	1.23	0.76	0.54	0.67	**1.30**	0.76	0.79
		GUG	**1.68**	0.56	0.66	0.82	1.08	0.85	0.75	1.04	1.02
(5)	Serine (S)	UCU	0.00	1.03	1.38	1.47	1.03	1.45	1.57	1.49	1.48
		UCC	0.00	0.21	0.40	0.78	0.24	0.68	0.71	0.53	0.55
		UCA	**4.00**	1.67	**1.88**	1.30	1.66	**1.90**	**1.97**	**1.66**	**1.69**
		UCG	0.00	0.47	0.13	0.00	0.06	0.48	0.31	0.32	0.31
		AGU	2.00	1.13	1.04	**1.61**	**1.93**	0.79	1.00	0.93	0.91
		AGC	0.00	**1.49**	1.17	0.83	1.07	0.71	0.45	1.07	1.05
(6)	Proline (P)	CCU	0.75	**1.93**	**1.79**	1.44	1.16	**1.32**	**1.89**	**1.35**	**1.29**
		CCC	0.46	0.62	0.76	0.30	0.50	0.99	0.63	0.98	1.02
		CCA	**1.86**	0.94	1.20	**1.66**	**1.50**	1.11	1.05	1.24	1.18
		CCG	0.93	0.51	0.25	0.60	0.84	0.57	0.43	0.44	0.50
(7)	Threonine (T)	ACU	1.00	1.12	1.02	1.00	**1.80**	**1.80**	1.49	**1.80**	**1.78**
		ACC	1.12	0.49	1.16	1.33	0.84	0.74	0.52	0.67	0.62
		ACA	**1.21**	**2.11**	**1.63**	**1.63**	1.36	1.32	**1.70**	1.53	1.60
		ACG	0.67	0.29	0.20	0.04	0.00	0.14	0.30	0.00	0.00
(8)	Alanine (A)	GCU	0.24	1.39	1.30	**1.79**	**1.77**	1.07	**1.53**	1.30	1.31
		GCC	1.76	0.65	0.51	1.27	0.62	0.32	0.70	0.29	0.35
		GCA	**2.00**	**1.86**	**1.63**	0.88	1.47	**2.13**	1.45	**2.41**	**2.34**
		GCG	0.00	0.10	0.56	0.07	0.14	0.48	0.32	0.00	0.00
(9)	Tyrosine (Y)	UAU	**1.33**	**1.30**	0.96	0.76	**1.36**	0.95	**1.24**	0.73	0.71
		UAC	0.67	0.70	**1.04**	**1.24**	0.64	**1.05**	0.76	**1.27**	**1.29**
(10)	Histidine (H)	CAU	**2.00**	**1.69**	0.93	**1.01**	**1.00**	0.95	**1.25**	**1.20**	**1.35**
		CAC	0.00	0.31	**1.07**	0.99	1.00	**1.05**	0.75	0.80	0.65
(11)	Glutamine (Q)	CAA	0.83	**1.16**	**1.31**	0.87	**1.15**	**1.23**	**1.39**	**1.24**	**1.23**
		CAG	**1.17**	0.84	0.69	**1.13**	0.85	0.77	0.61	0.76	0.77
(12)	Asparagine (N)	AAU	0.67	**1.14**	**1.47**	0.94	**1.22**	**1.23**	**1.35**	1.00	1.00
		AAC	**1.33**	0.86	0.53	**1.06**	0.78	0.77	0.65	1.00	1.00
(13)	Lysine (K)	AAA	0.88	**1.19**	**1.36**	0.84	**1.08**	**1.12**	**1.20**	**1.20**	**1.18**
		AAG	**1.12**	0.81	0.64	**1.16**	0.92	0.88	0.80	0.80	0.82
(14)	Aspartic acid (D)	GAU	**1.50**	**1.14**	0.86	**1.57**	**1.03**	**1.26**	**1.33**	**1.31**	**1.32**
		GAC	0.50	0.86	**1.14**	0.43	0.97	0.74	0.67	0.69	0.68
(15)	Glutamic acid (E)	GAA	0.87	**1.09**	**1.02**	0.69	**1.18**	**1.25**	**1.13**	**1.20**	**1.24**
		GAG	**1.13**	0.91	0.98	**1.31**	0.82	0.75	0.87	0.80	0.76
(16)	Cysteine (C)	UGU	**1.13**	**1.14**	**1.24**	**1.87**	0.00	**1.18**	**1.33**	**1.09**	**1.13**
		UGC	0.87	0.86	0.76	0.13	0.00	0.82	0.67	0.91	0.87
(17)	Arginine (R)	CGU	0.00	0.00	0.58	0.61	0.32	0.38	0.56	0.60	0.64
		CGC	0.00	0.00	0.00	0.27	0.00	0.39	0.02	0.00	0.00
		CGA	0.55	1.03	0.49	0.71	0.29	0.86	0.56	1.25	1.18
		CGG	0.00	0.04	0.10	0.39	0.27	0.52	0.07	0.50	0.58
		AGA	**3.47**	**3.02**	**3.68**	**2.24**	**3.50**	**2.92**	**3.33**	**2.35**	**2.39**
		AGG	1.98	1.90	1.16	1.78	1.63	0.92	1.46	1.30	1.21
(18)	Glycine (G)	GGU	0.57	1.17	0.96	0.97	0.61	0.52	1.12	0.29	0.32
		GGC	0.00	0.37	0.32	0.82	0.86	0.60	0.41	0.73	0.69
		GGA	0.94	1.21	**1.63**	**1.25**	**1.73**	**2.08**	**1.24**	**2.43**	**2.47**
		GGG	**2.49**	**1.25**	1.08	0.96	0.80	0.80	1.24	0.55	0.52

*The values of preferred codon for different coding sequences have been shown bold and highlighted.*

**Table 2:** Average RSCU values of the codons of the Nipah Virus **(**NiV) genome and comparison with the RSCU values of its natural host as well as experimental animal model hosts.

Amino acid	Codons	Average RSCU values										
		NiV	*Homo sapiens*	*Pteropus vampyrus*	*Equus caballus*	*Canis lupus*	*Felis catus*	*Mesocricetus auratus*	*Sus scrofa*	*Mustela putorius*	*Saimiri sciureus*	*Chloroceb aethiops*
**F**	UUU	**1.146**	0.93	0.49	0.83	0.4	0.81	0.81	0.78	0.71	0.68	0.89
	UUC	0.854	**1.07**	**1.01**	**1.16**	**1.59**	**1.18**	**1.18**	**1.21**	**1.28**	**1.31**	**1.1**
**L**	UUA	0.722	0.46	0.44	0.32	0.16	0.35	0.32	0.31	0.26	0.26	0.41
	UUG	1.221	0.77	0.8	0.72	0.38	0.75	0.79	0.67	0.63	0.6	0.74
	CUU	0.963	0.79	0.85	0.73	0.46	0.66	0.75	0.65	0.55	0.5	0.91
	CUC	0.740	1.17	**1.22**	1.31	1.55	1.28	1.21	1.34	1.55	1.44	1.15
	CUA	1.093	0.43	0.44	0.34	0.28	0.35	0.41	0.33	0.38	0.32	0.43
	CUG	**1.262**	**2.37**	0.37	**2.55**	**3.15**	**2.57**	**2.49**	**2.67**	**2.56**	**2.85**	**2.33**
**I**	AUU	**1.164**	1.08	1.19	0.9	0.76	0.94	1.01	0.9	0.82	0.8	1.08
	AUC	1.066	**1.41**	**1.3**	**1.65**	**1.76**	**1.58**	**1.57**	**2.34**	**1.85**	**1.77**	**1.46**
	AUA	0.770	0.51	0.49	0.42	0.47	0.47	0.41	0.42	0.38	0.42	0.45
**V**	GUU	**1.455**	0.72	0.72	0.6	0.22	0.62	0.6	0.56	0.39	0.57	0.8
	GUC	0.685	0.96	0.97	1.07	1.06	1.12	1.03	1.06	1.34	1.08	1
	GUA	0.919	0.47	0.51	0.35	0.38	0.38	0.42	0.33	0.3	0.3	0.43
	GUG	0.942	**1.85**	**1.79**	**1.96**	**2.31**	**1.87**	**1.93**	**2.03**	**1.95**	**2.03**	**1.76**
**S**	UCU	1.211	0.91	1.03	1.08	0.81	1.11	1.15	0.99	0.87	1.03	1.25
	UCC	0.455	1.09	1.22	1.43	2.01	1.48	1.39	1.5	1.65	1.54	1.25
	UCA	**1.970**	0.90	0.89	0.79	0.66	0.74	0.8	0.72	0.58	0.73	0.93
	UCG	0.231	**1.44**	0.29	0.33	0.22	0.38	0.3	0.38	0.42	0.34	0.28
	AGU	1.260	1.12	0.98	0.85	0.63	0.8	0.89	0.77	0.75	0.73	0.9
	AGC	0.872	1.31	**1.56**	**1.48**	**1.63**	**1.47**	**1.44**	**1.62**	**1.7**	**1.61**	**1.36**
**P**	CCU	**1.436**	0.90	1.21	1.19	0.83	1.02	1.18	1.04	1	1.04	1.08
	CCC	0.696	0.33	1.21	**1.37**	**1.85**	**1.5**	**1.3**	**1.45**	**1.45**	**1.39**	**1.27**
	CCA	1.304	1.15	**1.23**	0.97	0.83	0.96	1.14	0.94	0.87	0.92	1.27
	CCG	0.564	**1.30**	0.35	0.45	0.46	0.5	0.36	0.55	0.66	0.63	0.35
**T**	ACU	1.422	1.11	0.97	0.93	0.55	0.84	0.95	0.83	0.71	0.85	0.94
	ACC	0.832	0.45	**1.42**	**1.58**	**1.89**	**1.59**	**1.53**	**1.68**	**1.8**	**1.77**	**1.28**
	ACA	**1.565**	0.98	1.23	0.95	1.13	0.93	1.1	0.91	0.81	0.87	1.17
	ACG	0.182	**1.42**	0.37	0.52	0.41	0.62	0.4	0.57	0.66	0.49	0.3
**A**	GCU	1.299	1.14	1.12	1.05	0.87	0.94	1.15	0.95	0.88	1.03	1.21
	GCC	0.719	0.46	**1.57**	**1.72**	**2.11**	**1.8**	**1.62**	**1.8**	**1.9**	**1.75**	**1.56**
	GCA	**1.797**	1.06	0.94	0.77	0.61	0.75	0.86	0.73	0.69	0.75	0.89
	GCG	0.184	**1.60**	0.35	0.45	0.4	0.49	0.34	0.5	0.51	0.45	0.32
**Y**	UAU	**1.038**	**0.91**	0.87	0.75	0.74	0.78	0.83	0.73	0.77	0.72	0.93
	UAC	0.962	0.43	**1.12**	**1.25**	**1.25**	**1.21**	**1.16**	**1.26**	**1.22**	**1.27**	**1.06**
**H**	CAU	**1.264**	0.55	0.81	0.8	0.47	0.74	0.79	0.7	0.68	0.65	0.81
	CAC	0.736	**1.28**	**1.18**	**1.19**	**1.52**	**1.25**	**1.2**	**1.29**	**1.31**	**1.34**	**1.1**
**Q**	CAA	**1.157**	0.76	0.49	0.51	0.39	0.55	0.46	0.44	0.53	0.52	0.6
	CAG	0.843	**1.40**	**1.5**	**1.48**	**1.6**	**1.44**	**1.53**	**1.55**	**1.46**	**1.47**	**1.39**
**N**	AAU	**1.113**	**1.01**	0.92	0.83	0.62	0.82	0.81	0.78	0.75	0.76	0.94
	AAC	0.887	0.99	**1.07**	**1.16**	**1.37**	**1.17**	**1.18**	**1.21**	**1.25**	**1.23**	**1.05**
**K**	AAA	**1.117**	0.89	0.84	0.79	0.66	0.86	0.71	0.76	0.73	0.75	0.91
	AAG	0.883	**1.11**	**1.15**	**1.2**	**1.33**	**1.13**	**1.28**	**1.23**	**1.26**	**1.25**	**1.08**
**D**	GAU	**1.259**	0.84	0.98	0.83	0.62	0.84	0.81	0.8	0.76	0.74	0.97
	GAC	0.741	**1.16**	**1.01**	**1.16**	**1.37**	**1.15**	**1.18**	**1.19**	**1.23**	**1.25**	**1.02**
**E**	GAA	**1.073**	0.53	**1.88**	0.76	0.57	0.86	0.77	0.72	0.74	0.75	0.85
	GAG	0.927	**1.47**	1.05	**1.23**	**1.42**	**1.13**	**1.22**	**1.27**	**1.25**	**1.24**	**1.14**
**C**	UGU	**1.122**	0.94	0.92	0.89	0.77	0.86	0.85	0.78	0.66	0.73	0.91
	UGC	0.655	**1.06**	**1.07**	**1.1**	**1.22**	**1.13**	**1.14**	**1.21**	**1.33**	**1.26**	**1.08**
**R**	CGU	0.410	0.87	0.49	0.54	0.5	0.41	0.55	0.44	0.38	0.43	0.54
	CGC	0.075	1.13	0.94	1.15	1.76	1.09	1.08	**1.31**	**1.35**	1.35	1.12
	CGA	0.768	0.93	0.74	0.6	0.5	0.55	0.71	0.6	0.64	0.63	0.64
	CGG	0.274	1.07	1.18	1.08	1.41	1.19	1.11	1.28	1.27	1.08	1.05
	AGA	**2.991**	0.85	1.26	1.29	0.86	1.33	1.25	1.11	1	1.06	**1.44**
	AGG	1.482	**1.15**	**1.36**	**1.31**	**0.94**	**1.41**	**1.26**	1.23	1.33	**1.42**	1.18
**G**	GGU	0.727	0.65	0.71	0.64	0.65	0.58	0.7	0.56	0.57	0.64	0.6
	GGC	0.533	**1.35**	**1.36**	**1.42**	**2.06**	**1.42**	**1.29**	**1.46**	**1.44**	**1.51**	**1.24**
	GGA	**1.663**	1.00	0.96	0.95	0.39	1.01	1.01	0.91	0.88	0.91	1.24
	GGG	1.077	1.00	0.95	0.96	0.88	0.98	0.97	1.05	1.08	0.92	0.9

*The RSCU value of preferred codon have been shown bold and highlighted.*

**FIGURE 1:**
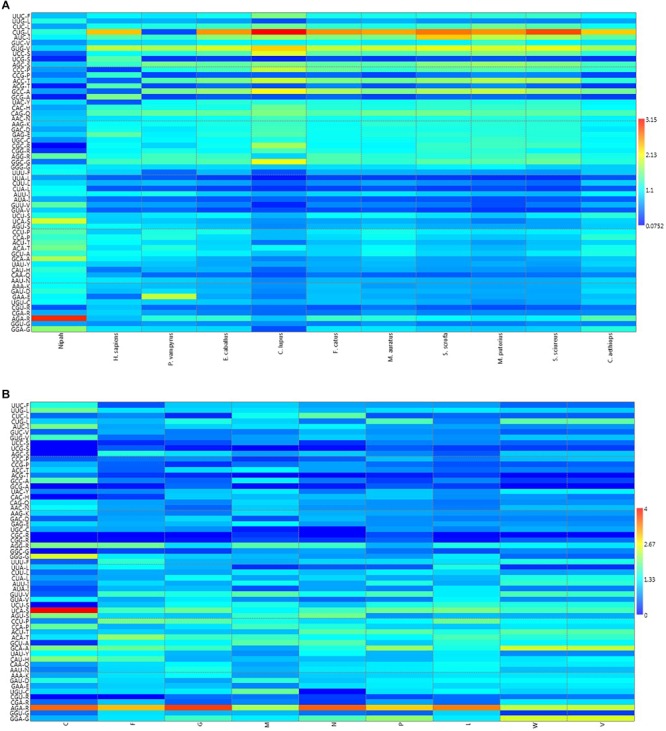
**(A)** Heat map of RSCU values (averages) of NiV and its plausible hosts. **(B)** Heat map comparing average RSCU values for different CDSs of NiV. The map indicates that, within genes themselves, codon preferences differ (higher RSCU values representing more frequent codon usage depicted in dark red, and lower RSCU values depicted in dark blue).

A heat map of RSCU values of various NiV strains and its hosts was constructed (Figure 1A); which revealed that NiV codon preference differed from that of the NiV hosts, with the exceptions of Leu, Tyr, and Asn. In the case of Leu, NiV, and *P. vampyrus* showed similar codon preferences; whereas for Tyr and Asn, NiV and *H. sapiens* showed similar codon preferences. Different preferences in codon usage by NiV and its hosts indicate that the virus does not compete for the host tRNA pool. The heat map of RSCU values for all the nine CDSs of NiV and hosts indicated that A/U-ending codons were preferred over G/C-ending codons (Figure 1B).

### Variable Intra-Genic Codon Usage Bias in NiV CDSs

Nc values were calculated to determine intra-genic codon bias. Within genes, the Nc value ranged from 48.8 to 56.2, with a mean value of 51.57 ± 1.64 (Supplementary Table S2). The highest Nc value was 56.36 ± 1.69 for the M gene, whereas the lowest value was 49.54 ± 0.78 for the C gene. The Tukey HSD test at a 95% confidence level revealed that there was a statistically significant difference in the mean Nc values between the genes (C-G, C-M, C-P, C-W, C-V, F-G, F-M, F-P, F-W, F-V, G-M, G-P, G-L, G-V, M-N, M-P, M-L, M-W, M-V, N-P, N-L, N-W, N-V, P-L, L-W, and L-V). Overall, the higher Nc values (>35) revealed little bias in codon usage.

### Nc-GC3 Curve Analysis

**FIGURE 2:**
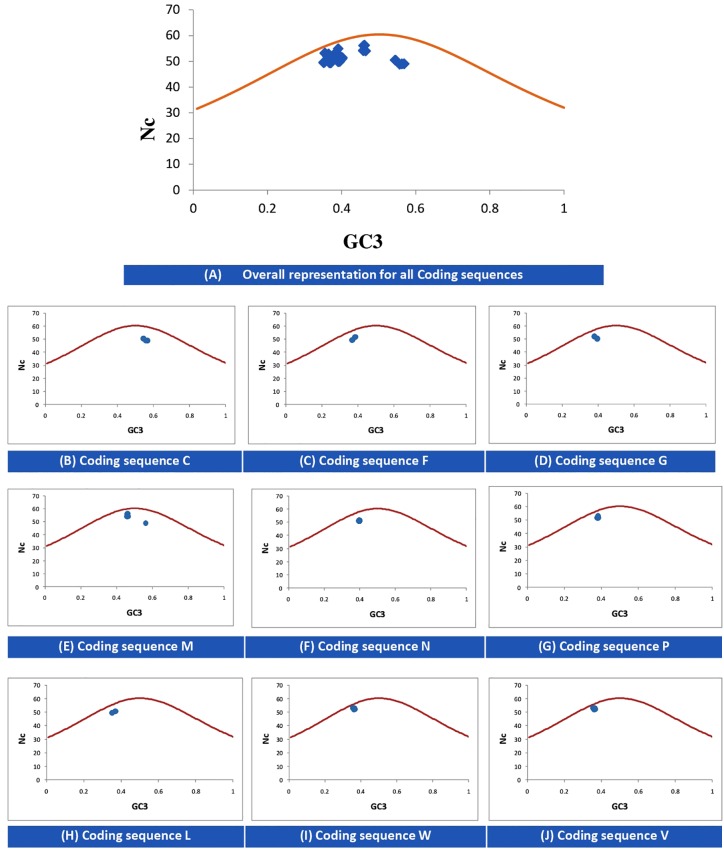
Nc–GC3 plots of different genes of NiV. The Nc curve is indicating the expected codon usage, if GC compositional constraints only account for the codon usage bias. Panel **(A)** for all coding sequences; **(B–J)** for coding sequence C, F, G, M, N, P, L, W, and V, respectively.

The Nc-GC3s plot is used to determine whether the codon usage of given genes is solely due to mutational pressure or selectional pressure. If the data points fall onto the expected curve, it indicates mutational pressure; whereas if the points fall below the expected curve, the codon usage is said to be affected by selectional pressure. Figure 2 indicates that for all CDSs, selectional pressure is the major force influencing the codon usage in NiV.

### Neutrality Plot

**FIGURE 3:**
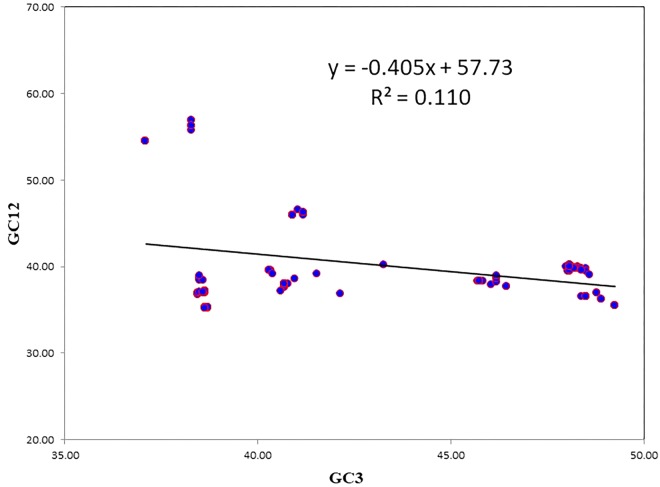
The neutrality plot to analyze the influences of mutation bias and translation selection on codon usage. GC12 stands for the average value of GC content at first and second position of codon. GC3 stands for GC content at third position of codon. The slope value indicates the mutational pressure.

A neutrality plot between GC12 and GC3 was used to reveal the degree of mutational pressure on the codon usage. In synonymous codons, only the last nucleotide is different, and the amino acid remained unaltered. Because nucleotide changes at the third position of the codon does not contribute to changes in the amino acid, it is indicative of a mutational force only. When nucleotide change brings about change in the altered amino acid, it leads to selection force. When there is a correlation between the GC12 and GC3, it is likely to be due to mutational forces, since the force influencing codon bias is present at all the codon positions (Jenkins and Holmes, 2003). The average value of GC12 was negatively correlated with the average GC3 (*r* = −0.333331; *P* < 0.001). The results of the neutrality plot revealed statistically significant correlations between GC12 and GC3, however, the slope value was less, indicating the role of mutational pressure, though not as the dominant pressure (Figure 3). The slope of the regression line was 0.405, which suggests that the relative neutrality (mutation pressure) was 40.5% and that the relative constraint on the GC3 (natural selection) was 59.5%. The neutrality plot indicated that selection pressure dominated over mutational pressure.

### Parity Analysis

The vector from the center represents the extent and direction of biases from PR2. PR2 bias plots are particularly informative when PR2 biases at the third codon position in four-codon sequences of individual genes are plotted. As per Chargaff’s second parity rule (PR2), the number of residues A = T and residues C = G in a DNA strand (Rapoport and Trifonov, 2013). The center of the plot, where both coordinates are 0.5, is the place where A = T and G = C (PR2), or in other words, it is the place where no bias is present in the selection (substitution rates) or mutation force in complimentary strands of DNA (Sueoka, 1988).

**FIGURE 4:**
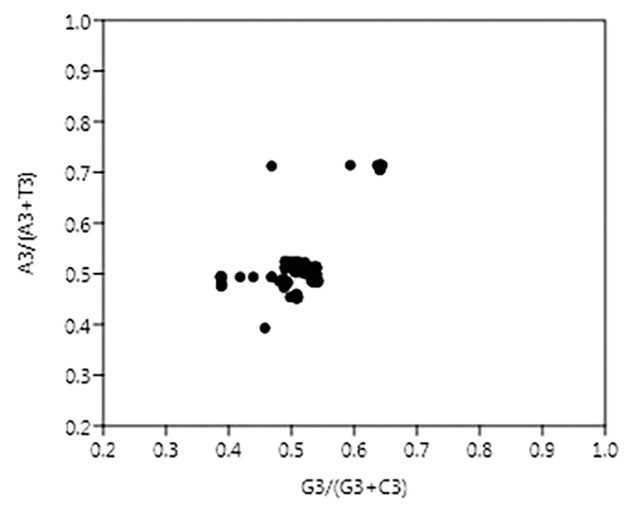
Parity plot showing the presence of AT bias [A3%/(A3% + T3%)] and GC bias [G3%/(G3% + C3%)]. The center of the plot, where value of both the coordinates is 0.5, indicates the place where there is no bias in mutation or selection rates.

In this case, A3/(A3 + T3) and G3/(G3 + C3) were plotted as the ordinate and abscissa, respectively (Figure 4). The mean value of AT bias [A3/(A3 + T3)] was 0.51 and GC bias [G3/(G3 + C3)] was 0.511. A value of bias more than 0.5 indicates the preference of purine over pyrimidine (Zhang et al., 2018). Hence, here A will be preferred over T and likewise G will be preferred over C.

### Isoacceptor tRNA Pool

Different isoacceptor tRNAs of various tRNA species bind to different codons that signify a particular amino acid residue. Table 3 shows the frequency of tRNA genes in human cells; for a single codon, a variable number of isoacceptor tRNAs are present in a cell and these are different for different species. Whether most codons preferred by NiV are recognized by most abundant isoacceptor tRNAs, determines translation selection (Kumar et al., 2016). Out of 18 amino acids (which are encoded by two or more amino acids) except for Leu, Ile, Val, and Pro, all non-optimal codon-anticodon base pairs were used (Table 3).

**Table 3:** Frequency of tRNA genes in human cells for most preferentially used codons in Nipah virus (NiV).

Amino acid	Most preferred codons in NiV	tRNA isotypes in human cells	Total count
Ala (A)	GCA	AGC (22), GGC (0), CGC (4), UGC (8)	34
Gly (G)	GGA	ACC (0), GCC (14), CCC (5), UCC (9)	28
Pro (P)	CCU	AGG (9), GGG (0), CGG (4), UGG (7)	20
Thr (T)	ACA	AGU (9), GGU (0), CGU (5), UGU (6)	20
Val (V)	GUU	AAC (9), GAC (0), CAC (11), UAC (5)	25
Ser (S)	UCA	AGA (9), GGA (0), CGA (4), UGA (4), ACU (0), GCU (8)	25
Arg (R)	AGA	ACG (7), GCG (0), CCG (4), UCG (6), CCU (5), UCU (6)	28
Leu (L)	CUG	AAG (9), GAG (0), CAG (9), UAG (3), CAA (6), UAA (4)	31
Phe (F)	UUU	AAA (0), GAA (10)	10
Asn (N)	AAU	AUU (0), GUU (20)	20
Lys (K)	AAA	CUU (15), UUU (12)	27
Asp (D)	GAU	AUC (0), GUC (13)	13
Glu (E)	GAA	CUC (8), UUC (7)	15
His (H)	CAU	AUG (0), GUG (10)	10
Gln (Q)	CAA	CUG (13), UUG (6)	19
Ile (I)	AUU	AAU (14), GAU (3), UAU (5)	22
Tyr (Y)	UAU	AUA (0), GUA (13)	13
Cys (C)	UGU	ACA (0), GCA (29)	29
Trp (W)	UGG	CCA (7)	7
Met (M)	AUG	CAU (9/10)	19

### Measures of Virus Adaptation (CAI and RCDI)

Codon adaptation index values are used to determine the level of expression of pathogen proteins in the host and adaptation of a virus to a host. Sequences with higher CAI values are considered more adapted to particular hosts than those with low values. The CAI values were 0.75 ± 0.019, 0.74 ± 0.017, 0.72 ± 0.017, 0.67 ± 0.021, 0.66 ± 0.02, 0.65 ± 0.027, 0.65 ± 0.021, 0.61 ± 0.022, 0.59 ± 0.022, and 0.58 ± 0.026 for the African green monkey, bat, human, hamster, dog, horse, cat, pig, squirrel monkey, and ferret, respectively (Table 4). The comparative analysis based on CAI values indicated that NiV host adaptation was greatest for the African green monkey, followed by the bat and human; while it was least for ferrets. A comparative analysis of CAI for HeV, CedV, and MojV among different hosts considered for the study, revealed that these viruses are highly adapted in African green monkeys as was observed in case of NiV, but is least adapted in dogs (Table 4).

**Table 4:** List of a total of 10 hosts for Nipah virus (NiV), Hendra virus (HeV), Cedar virus (CedV), and Hendra like Mojiang virus (MojV), the details of number of coding sequences (CDSs), and codons used to calculate CAI values.

S. no.	Species (Common name)	Number of CDSs evaluated	Total number of codons	Average CAI value ± *SD*			
				NiV	HeV	CedV	MojV
(1)	*Homo sapiens* (Human)	19250	11090056	0.728 ± 0.017	0.738 ± 0.02	0.746 ± 0.017	0.734 ± 0.019
(2)	*Pteropus vampyrus* (Bat)	261	138222	0.749 ± 0.018	0.746 ± 0.018	0.757 ± 0.018	0.745 ± 0.019
(3)	*Equus caballus* (Horse)	420	156469	0.653 ± 0.022	0.649 ± 0.021	0.666 ± 0.021	0.649 ± 0.024
(4)	*Canis familiaris* (Dog)	1194	559501	0.66 ± 0.021	0.497 ± 0.021	0.501 ± 0.025	0.497 ± 0.027
(5)	*Sus scrofa* (Pig)	2953	1168059	0.613 ± 0.022	0.612 ± 0.023	0.627 ± 0.021	0.611 ± 0.025
(6)	*Felis catus* (Cat)	362	139977	0.657 ± 0.022	0.656 ± 0.022	0.668 ± 0.020	0.661 ± 0.011
(7)	*Mesocricetus auratus* (Hamster)	330	141900	0.674 ± 0.022	0.663 ± 0.021	0.685 ± 0.021	0.668 ± 0.022
(8)	*Mustela putorius* (Ferret)	46	19006	0.581 ± 0.026	0.576 ± 0.027	0.597 ± 0.026	0.579 ± 0.030
(9)	*Saimiri sciureus* (Squirrel Monkey)	55	17382	0.597 ± 0.023	0.591 ± 0.024	0.610 ± 0.022	0.593 ± 0.027
(10)	*Chlorocebus aethiops* (African Green Monkey)	167	73417	0.755 ± 0.019	0.750 ± 0.022	0.764 ± 0.019	0.748 ± 0.020

*Highest values have been highlighted green; whereas the lowest highlighted as orange.*

There was a strong correlation between CAI and Nc (*r* = 0.513; *P* < 0.0001). A correlation analysis between GC3%, Nc, CAI, GRAVY, and AROMO, is provided in Table 5. GRAVY and AROMO are indexes of natural selection influencing codon bias, where GC3 is an indicator of compositional properties (Barbhuiya et al., 2019). GC3 is positively correlated with CAI and AROMO, while negatively correlated with GRAVY; which indicates that with an increasing compositional bias, aromaticity and gene expression are increased while hydropathy is decreased.

**Table 5:** Correlation analysis between GC3%, Nc, CAI, GRAVY, and AROMO.

	GC3%	Nc	CAI	GRAVY
Nc	0.026			
CAI	0.635^∗∗∗^	0.513^∗∗∗^		
GRAVY	−0.410^∗∗∗^	−0.016	0.004	
AROMO	0.294^∗∗∗^	−0.048	0.017	−0.741^∗∗∗^

*^∗∗∗^p < 0.01.*

The correlation analysis between CAI, GRAVY, and AROMO was done to determine the effect of GRAVY and AROMO (indicator of natural selection) on expressivity of gene (indicated by CAI), wherein no correlation was obtained.

**FIGURE 5:**
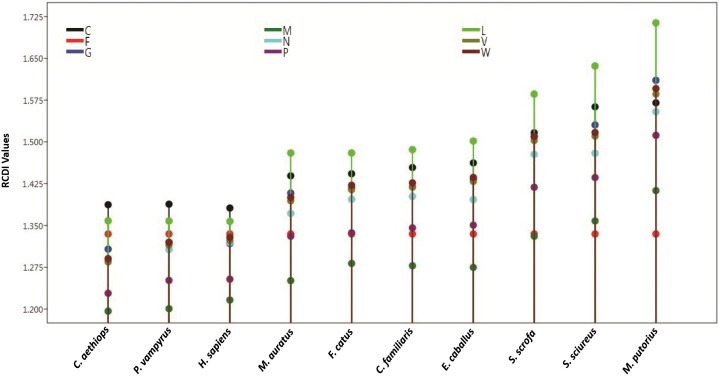
Tree plot for intragenic RCDI analysis. The plot indicates an average RCDI values with reference to different hosts and the 9 coding sequences of NiV. For adaptation of NiV genome, with least RCDI values African green monkey and with highest RCDI values Ferrets are best and least adapted, respectively, for growth NiV, where exhibiting the RCDI and hence east adapted. (For a single gene, an average RCDI has been taken).

Relative codon deoptimization index (RCDI) values indicate the cumulative effects of codon biases on the expression of a gene. It is measured by comparing the codon usage of a virus with that of its host. These values also provide insight into the possible co-evolution of virus and host genomes. A lower RCDI value indicates higher adaptation of a virus to its host. Our intragenic RCDI analysis revealed that for NiV, in all tested hosts, except for the squirrel monkey and ferret, the M gene showed the lowest RCDI, indicating a high degree of adaptation to these hosts (Table 6). The L gene had the highest RCDI value for all the hosts except the human, bat, and African green monkey, for which the maximum RCDI values were observed in the C gene. Average RCDI values revealed that NiV was best adapted to the African green monkey (RCDI value of 1.297), followed by the bat (1.311) and human (1.315). Ferrets showed the highest RCDI value (average 1.543; Figure 5), indicating poor adaptation. A higher adaptation will naturally increase the infectivity and vice versa. This observation is corroborated by the comparison of CAI values. Average RCDI values of other viruses in different hosts revealed that among the evaluated hosts and viruses, except for Hamster and Dog, HeV is more adapted than NiV, CedV, and MojV. On the basis of the CedV RCDI value, it appears that this virus is least adapted. However, this needs to be confirmed through an analysis of a greater number of sequences, so as to be statistically more pertinent.

**Table 6:** RCDI values of various coding sequences (CDSs) of Nipah virus (NiV), Hendra virus (HeV), Cedar virus (CedV), and Hendra like Mojiang virus (MojV) in different hosts.

Name of host	Virus type	CDSs									Average RCDI
		C	F	G	M	N	P	L	V	W	
*H. sapiens*	NiV	1.381	1.335	1.317	1.216	1.319	1.254	1.357	1.323	1.329	1.315
	HeV	1.407	1.266	1.259	1.199	1.274	1.251	1.343	1.301	1.301	**1.289**
	CedV	1.594	1.466	1.427	1.348	1.324	1.244	1.364	–	–	**1.395**
	MojV	1.411	1.418	1.292	1.386	1.284	1.324	1.352	–	–	1.352
*P. vampyrus*	NiV	1.388	1.335	1.320	1.201	1.307	1.251	1.358	1.315	1.320	1.311
	HeV	1.418	1.264	1.250	1.187	1.255	1.251	1.339	1.302	1.303	**1.286**
	CedV	1.629	1.458	1.428	1.356	1.310	1.243	1.368	–	–	**1.399**
	MojV	1.426	1.398	1.297	1.378	1.268	1.309	1.351	–	–	1.347
*E. caballus*	NiV	1.462	1.335	1.433	1.275	1.396	1.351	1.502	1.429	1.436	1.402
	HeV	1.471	1.376	1.355	1.265	1.341	1.349	1.477	1.403	1.403	**1.382**
	CedV	1.691	1.633	1.604	1.460	1.426	1.335	1.517	–	–	**1.524**
	MojV	1.472	1.602	1.420	1.503	1.385	1.456	1.509	–	–	1.478
*C. familiaris*	NiV	1.454	1.335	1.419	1.278	1.402	1.346	1.486	1.419	1.426	**1.396**
	HeV	1.820	1.796	1.754	1.582	1.722	1.860	1.958	1.966	1.964	1.825
	CedV	2.029	2.083	2.105	1.915	1.929	1.720	2.074	–	–	**1.979**
	MojV	1.770	2.039	1.771	1.969	1.784	1.885	2.096	–	–	1.902
*S. scrofa*	NiV	1.516	1.335	1.509	1.331	1.478	1.419	1.586	1.502	1.510	1.465
	HeV	1.516	1.434	1.428	1.321	1.420	1.410	1.561	1.474	1.474	**1.449**
	CedV	1.737	1.721	1.691	1.519	1.504	1.398	1.597	–	–	**1.595**
	MojV	1.513	1.681	1.487	1.574	1.457	1.522	1.588	–	–	1.546
*F. catus*	NiV	1.443	1.335	1.416	1.282	1.397	1.337	1.480	1.414	1.422	1.392
	HeV	1.433	1.362	1.355	1.269	1.346	1.340	1.458	1.399	1.399	**1.373**
	CedV	1.650	1.605	1.588	1.428	1.414	1.309	1.486	–	–	**1.497**
	MojV	1.444	1.491	1.407	1.491	1.384	1.435	1.486	–	–	1.448
*M. auratus*	NiV	1.439	1.335	1.408	1.251	1.372	1.331	1.480	1.394	1.400	**1.379**
	HeV	1.457	1.353	1.328	1.236	1.320	1.327	1.455	1.378	1.378	1.359
	CedV	1.705	1.605	1.579	1.461	1.394	1.315	1.507	–	–	**1.509**
	MojV	1.465	1.539	1.399	1.481	1.340	1.418	1.488	–	–	1.447
*M. putorius*	NiV	1.570	1.335	1.611	1.413	1.554	1.512	1.714	1.586	1.596	1.543
	HeV	1.551	1.538	1.519	1.378	1.496	1.495	1.674	1.575	1.575	**1.533**
	CedV	1.840	1.864	1.853	1.611	1.608	1.459	1.729	–	–	**1.709**
	MojV	1.544	1.825	1.594	1.687	1.551	1.622	1.733	–	–	1.651
*S. sciureus*	NiV	1.563	1.335	1.530	1.358	1.480	1.436	1.636	1.510	1.517	1.485
	HeV	1.554	1.457	1.458	1.349	1.426	1.444	1.608	1.507	1.507	**1.479**
	CedV	1.419	1.777	1.758	1.558	1.537	1.419	1.653	–	–	**1.589**
	MojV	1.517	1.741	1.513	1.613	1.483	1.560	1.653	–	–	1.583
*C. aethiops*	NiV	1.387	1.335	1.308	1.196	1.287	1.228	1.358	1.285	1.291	1.297
	HeV	1.394	1.268	1.251	1.185	1.248	1.246	1.335	1.284	1.284	**1.277**
	CedV	1.600	1.488	1.445	1.358	1.302	1.223	1.369	–	–	**1.398**
	MojV	1.424	1.443	1.303	1.400	1.267	1.321	1.361	–	–	1.360

*Highest values have been shown bold and highlighted blue and lowest as orange.*

A correlation analysis of CAI values with RCDI values of different hosts revealed a significant negative correlation (*p* < 0.01; *r* = −0.227 to −0.411). A candidate virus genome rationally designed through modified codon usage possessing lower CAI and higher RCDI; that will result in production of viral proteins at a lower level, leading to decreased viral infectivity, i.e., a step closer to the development of an attenuated vaccine candidate. A comparative analysis of RCDI values of all the viruses in various hosts, indicates that except for *Canis familiaris* and *Mesocricetus auratus*, HeV is more adapted than NiV in all the hosts (Table 6).

### Similarity Index

To determine the role of a host in shaping codon usage by NiV, similarity indexes were calculated for all the hosts. This method allowed for a direct measurement of the similarities in codon usage between the pathogen and host, taking 59 codons as 59 spatial vectors. The values ranged between 0 and 1. The similarity indexes in the present investigation were 0.085, 0.062, 0.076, 0.139, 0.078, 0.070, 0.094, 0.104, 0.095, and 0.054 for the human, bat, horse, dog, cat, hamster, pig, ferret, squirrel monkey, and African green monkey, respectively. On the basis of these similarity indexes, the dog genome had the highest (0.139) impact on the NiV codon bias, followed by the ferret, whereas the African green monkey (0.054) had the lowest.

## Discussion

### Compositional Analysis

Overall, from initial nucleotide compositional analysis, the NiV genome is AU rich; and A/U ending codons appear to be preferred over G/C ending codons.

### The CpG Dinucleotide Is Depleted but Not TpA

Codon usage bias, mutational bias, as well as the influence of selective pressure and compositional constraints, are reflected in the occurrence of dinucleotides. The relative abundance of dinucleotides has shown to affect codon usage in RNA viruses(Belalov and Lukashev, 2013). CpG depletion is considered a selective force that affects the frequency of CpG-containing codons. The low relative abundance of CpG may be explained by three non-exclusive facts. First, unmethylated CpG-containing sequences are recognized as pathogen signatures by the innate defense systems of hosts; they stimulate immune responses. Thus, to evade host immunity, the virus may maintain low CpG content (Vetsigian and Goldenfeld, 2009). Second, lower CpG abundance may be linked to methylation of cytosine residues, an epigenetic change associated with important functions such as gene silencing, X chromosome inactivation, and genetic imprinting in germline cells (Li and Zhang, 2014). Methylated cytosines may be mutated into thymine through spontaneous deamination. This results in the formation of TpG instead of CpG, decreasing CpG content. NiV is an RNA virus, it showed an odds ratio of 0.258, which is within the range of odds ratios for most polyomaviruses, which have the lowest odds ratios for CpG dinucleotides among DNA viruses (Shackelton et al., 2006). Low CpG odds ratios are common in negative sense single-stranded viruses (Cheng et al., 2013). The RSCU values of all eight codons containing CpG (CCG, UCG, GCG, ACG, CGG, CGC, CGU, and CGA) were found to be underrepresented (RSCU < 0.78) indicative of the selection pressure acting on NiV in shaping codon usage. A similar underrepresentation of CpG-containing nucleotides has been observed in the genome of the equine influenza virus (Kumar et al., 2016).

Other dinucleotides that are commonly underrepresented in DNA and RNA virus genomes are TpA and UpA (Kumar et al., 2016). That is because of the higher susceptibility of UpA to cytoplasmic RNase, which helps maintain mRNA turnover in the cell. In addition, UpA avoidance may be related to its energetically unstable configuration (Breslauer et al., 1986), specifically when it is presented in a UUAUUUAU sequence that destabilizes mRNA (Beutler et al., 1989). UpA is available in two (out of the three) stop codons (UAA and UAG), so avoidance prevents non-sense mutations. In an echovirus-7 model, in which CpG/UpA dinucleotide frequency was artificially increased, the virus was attenuated, and its replication inhibited through an entirely unknown mechanism (Fros et al., 2017). However, in the case of NiV, surprisingly UpA frequency was not underrepresented, and the reason is attributed to A nucleotide rich genome. All six codons containing UpA (UUA, CUA, AUA, GUA, UAU, and UAC) had RSCU values >1.04, with odds ratios between 0.78 to 1.25 (1.18), indicating unbiased use of UpA-containing codons. This indicates that selectional pressure leading to low UpA frequencies is not actively involved in the codon usage patterns of NiV; rather, these patterns are mainly governed by compositional constraints, as the NiV genome is AU rich. This observation agrees with earlier observations of Kunec and Osterrieder (2016), who found that codon bias is essentially a direct consequence of dinucleotide bias.

### Multiple Factors Are Playing Role in Shaping Codon Usage

In the case of NiV, the average GC and GC3 content were 42.245 and 40.173, respectively. If codon usage is affected only by GC3 content, this indicates mutational pressure; in such cases, the Nc values lie just over the expected Nc curve (He et al., 2016). In all nine CDSs in the NiV genome, Nc values were below the expected Nc curve (Figure 2), i.e., Nc values were far from the curve, indicating the dominant role of selection pressure. Thereafter, we have also observed the role of selection pressure through the neutrality plot. When the correlation between GC12 and GC3 is statistically significant and the slope of the regression line is close to 1, mutational bias is assumed to be the main forces shaping the codon usage. Similarly, slopes reaching a value of 0 or a nearly horizontal line, are indicative of selection pressure being the dominant factor influencing codon usage. The neutrality plot again underscores the dominance of natural selection over mutational forces. The GC3 content in different CDSs varied widely, ranging from 35.2 to 56.9. In the neutrality plot, except for the M gene, all genes tended to remain far from the slope of the regression line (slope of regression line, *y* = –0.405*x* + 57.73, *R*^2^ = 0.110), further confirming the findings that mutational pressure is not the major factor affecting codon usage. Additionally, there was no correlation between Nc and GRAVY or Nc and AROMO, suggesting that codon usage bias is not affected by hydrophobicity or aromaticity. The correlation analysis between CAI, GRAVY, and AROMO was performed to determine the effect of GRAVY and AROMO (indicator of natural selection) on the expressivity of the gene (indicated by CAI values), which indicated that no correlation was observed. Parity analysis revealed that there is a bias for the third codon position which further reconfirms the role of selection pressure.

### Translational Selection

Most preferred codons of NiV uses suboptimal isoacceptor tRNAs. This can be explained by the fact that, during the initial phase of infection, when the translation rate is low, usage of suboptimal codons does not represent a constraint, whereas, during later stages of infection, the overall host cellular machinery is hijacked and compromised, allowing preferential translation of virus proteins. In both situations, usage of suboptimal codons has shown no detrimental effects on the translation of viral proteins (Mahajan and Agashe, 2018). In addition, deliberately suboptimal codons may be used to maintain a low level of translation to facilitate proper folding and the appropriate three-dimensional conformation of a nascent protein by introducing translational pauses, as in the case of the hepatitis A virus (Sánchez et al., 2003). All of these factors contribute to natural selection in NiV for efficient and accurate translation and proper folding of functional viral proteins (RoyChoudhury and Mukherjee, 2013).

The CAI values, RCDI values, and similarity indexes were evaluated and examined among all hosts. The African green monkey was found to be the best suited for animal experiments, with the highest CAI and similarity index; and the lowest RCDI value. CAI values are associated with selection pressure, with highly expressed codons selected. On the other hand, Nc is associated with a bias that is due to selection or mutation pressure. The correlation between CAI and Nc can be based on the relative balance between the selection and mutation (Vicario et al., 2007). A greater positive correlation (*r* = 0.513; *p* < 0.00001) indicates that a higher expression (indicated by CAI) is associated with increased bias (Nc) and that selection pressure, rather than mutational forces, are the dominant pressure in the NiV genome.

### Effects of Hosts on NiV Codon Usage

Similarity index analysis revealed that the dog genome and African green monkey has a maximum and minimum effect, respectively, on NiV codon usage. In comparison to the squirrel monkey, ferret, pig, and dog, human codon usage had less of an influence on NiV codon usage. Similarity indexes have been reported for other viruses, including chikungunya virus (Butt et al., 2014) and Zika virus (Butt et al., 2016). Our observations agree with the results of Wang et al. (2016), who observed similar indexes of *Aedes* *albopictus* or *Aedes* *aegypti* that were higher than those of humans for Zika virus. The relatively low average values of *D* (*A*, *B*) [where *D* (*A*, *B*), indicates the potential effects of the overall codon usage of the hosts on that of NiV] suggests that NiV can replicate efficiently in hosts without much of an effect on host codon usage. In the case of the Marburg virus, the host *Rousettus aegyptiacus* (Egyptian fruit bat) also showed a higher similarity index than for humans (Nasrullah et al., 2015).

### African Green Monkey Is the Most Adapted Host

Among the 10 tested hosts, ferrets were found to be the least supportive of NiV replication. Based on the intragenic analyses, clear statistically significant differences were observed in codon usage biases between the nine CDSs, and, along with compositional constraints, selectional forces were found to play a major role in shaping NiV codon usage. Deoptimization is measured by comparing the codon usage of a virus with that of its host. RCDI values also provide insight into the possible co-evolution of virus and host genomes. A lower RCDI value indicates higher adaptation of a virus to its host. The African Green monkey had lowest mean RCDI value (1.297 ± 3.282), indicating the highest adaptation. This result is in accordance with the fact that a cell line derived from the kidney epithelial cells of African green monkey (Vero cell line) is commonly employed for isolation of NiV. A study encompassing 10 cell lines was conducted to compare the susceptibility of cell lines to NiV and it was revealed that the virus replicated best in Vero cells and BSR cells (Aljofan et al., 2009).

A codon-optimized “humanized” green fluorescent protein has an RCDI of 1.31 (Mueller et al., 2006). A low RCDI value is often associated with a high CAI value. For polio virus, synthetic constructs with deoptimized synonymous codons in the capsid region were evaluated for their association with virus survival. Constructs with RCDI values <2 survived, while RCDI values >2 did not, indicating that viruses with higher RCDI values are not well adapted to a host (Mueller et al., 2006). However, a high RCDI value may reflect the expression of a few genes during latency or maintenance of a low translation rate to achieve error-proof translation and correct folding of viral proteins (Puigbo et al., 2010). Our data obtained from RCDI analysis is in accordance with experimental data of Johnston et al. (2015), who comprehensively and statistically evaluated the African green monkey model and found that disease progression parameters in the African green monkey were similar to and correlated with those in humans. Ferrets showed the highest RCDI value (average 1.543), indicating poor adaptation as well as the potential to be established successfully in a host with different codon usage patterns. Our results revealed that NIV is less adapted to ferrets; which is contradictory to the common observation that ferrets are a proven infection model for this virus, and that pathogenic symptoms are observed in this animal model. On the other hand, NiV is reported to be well adapted in bats, where it shows avirulence. Within host viral fitness and virulence are the commonly coupled phenomenon and should be positively correlated. However, certain mutations/genetic changes may exist, which may break this fitness-virulence relationship owing to the complex virus–host interactions. A quantification of viral fitness and virulence has been done for vesicular stomatitis virus (VSV) using 21 single- or double-nucleotide mutants in the BHK-21 cell line. Broadly speaking, a positive correlation has been found between these two phenomena, however, significant outliers (high fitness yet relatively low virulence and reduced fitness with no effects on virulence) have also been reported (Furió et al., 2012). This experiment of Furió et al. (2012) is able to explain our results, showing low calculated fitness, yet high virulence in a ferret model and vice versa in bats.

Our codon usage study though, does not exactly explain the amplitude of pathogenicity/virulence in animal models; however, it is able to predict the virus replication inside the host (even when the presence of virus is asymptomatic as in case of bat). Thus, it can easily predict the carrier hosts as well, which without being affected by virus, may act as a source of infection in other co-circulating species. The study was carried out with the aim to predict which animals may be possible hosts for NiV, whether they could directly show symptoms or be asymptomatic hosts. In all tested hosts used in the study, NiV is known to cause infection and some of these live in close vicinity to human populations in the rural areas. Hence, the present study was carried out to compare the adaptation of NiV in different hosts. The study will also pave the way to evaluate and assess other animals for their potential to serve as hosts for the virus. The information will be helpful in elucidating the emerging health hazards to humankind as a result of living in close contact with these animals, which may serve as carriers of the virus, although asymptomatic, could serve as a source of infection.

Our observation is corroborated with results obtained by comparing CAI values. NiV has recently emerged as a virus posing a major public health concern. There is an urgent need for the development of an effective vaccine and discovering viable therapeutics along with the identification of potential hosts. Information regarding potential hosts may be useful in identifying various preventive measures. Here, on the basis of codon usage, we attempted to identify hosts with the most potential to harbor the virus. The NiV sequences were found to be highly adapted to the bat and human sequences based on CAI values. Humans and bats were found to be highly supportive of virus replication, as evidenced by higher CAI values. Results of this study might also be useful in identifying the most suitable experimental animal models for vaccine and other pathogenicity-associated experiments.

Another advantage of the study can be linked to the development of a codon biased rationally designed synthetic attenuated virus vaccine candidate against NiV. For any attenuated virus vaccine candidate, genetic stability and safety are major concerns. Introduction of codon-pair bias de-optimization in hemagglutinin (HA) and neuraminidase (NA) gene of Influenza A virus resulted in the generation of a virus phenotype, that produces inappreciable pathogenesis, but at the same time elicited a robust immune response after its intranasal administration (Kaplan et al., 2018). Coleman et al. (2008) used the poliovirus as a model system to study the consequences of genome-wide codon deoptimization and achieved reduced poliovirus protein synthesis that resulted in virus attenuation. Replacement of codons with non-preferred synonymous codons in the capsid region of the rabies virus reduced the Nc value from 56.2 to 29.8 and the virus fitness (assessed by means of reduction in plaque size and virus yields) was also reduced in proportion to the number of replaced codons. Regarding the safety concern of such attenuated virus vaccines, deoptimized rabies virus constructs, have been demonstrated to retain most codon replacements along with replicative fitness that was quite lower than the wild type virus (Burns et al., 2006). The experiment of Burns et al. (2006) indicates that deoptimized vaccines may retain their replicative fitness without being reverted to previous genetic sequences. Reconstruction of an attenuated virus by codon deoptimization at genome-wide level containing hundreds to thousands of silent nucleotide mutations has a low risk of reversion, however, under selective pressure, the stability of such vaccine candidates is largely unknown. Research in this area suggests that deattenuation may progress slowly due to large scale accumulation of mutations, however, the deattenuation level is feeble. The same has been shown in the lymphocytic choriomeningitis virus (LCMV), where deoptimization in nucleoprotein (NP) gene resulted in a high degree of attenuation, but such a virus conferred 100% protection with a single dose of immunization. The aforementioned LCMV attenuated virus, exhibited the presence of only two silent point mutations (Cheng et al., 2017) upon 10 passages in Vero cells, showing that these viruses are genetically stable. In another experiment, the codon pair deoptimized human respiratory syncytial virus (RSV) vaccine candidate grown under gradually increasing temperature (32–40°C) for 17 passages was found to remain phenotypically and genotypically stable (Le Nouën et al., 2017). This is direct evidence that even under strong selection pressure, such a rationally designed synthetic virus is safe and less likely to reverse back to its original virulent phenotype. The information revealed, with regards to the codon bias of NiV in the present study, may be adapted similarly, to develop a codon deoptimized rationally designed vaccine in human vaccination programs.

## Conclusion

NiV has recently emerged as a virus that poses a major public health concern. There is an urgent need for the development of an effective vaccine and to discover viable therapeutics along with the identification of potential hosts. Information regarding potential hosts may be useful in identifying various preventive measures. Here, on the basis of codon usage, we attempted to identify hosts with the most potential in harboring the virus. Based on the intragenic analyses, there are clear statistically significant differences in codon usage bias between the nine CDSs, and, along with compositional constraints, selection forces were found to play a major role in shaping NiV codon usage. The NiV sequences were found to be highly adapted to the bat and human sequences based on CAI values. Humans and bats were found to be highly supportive of virus replication, as evidenced by higher CAI values. The results of this study might also be useful in identifying the most suitable experimental animal models for vaccine and other pathogenicity-associated experiments. After evaluating CAI values, RCDIs, and similarity indexes, among all hosts examined, the African green monkey was found to be the best suited for animal experiments, with highest CAI and similarity index and lowest RCDI value. Among the 10 tested hosts, ferrets were found to be the least supportive of NiV replication. Considering other viruses like HeV, CedV, and MojV, RCDI analysis indicated that except for ferrets and dogs, HeV is more adapted than NiV. The comparative analysis of CAI value indicated that the African green monkey is the most suitable animal model for studying *Henipavirus* (NiV, HeV, CedV, and MojV).

Various tools like DNAWorks, Jcat, Synthetic Gene Developer, GeneDesign, Gene Designer 2.0, OPTIMIZER, Visual Gene Developer, Eugene, mRNA Optimizer, Codon Optimization OnLine (COOL), and D-Tailor are freely available to optimize gene expression, without altering the amino acid sequence. These tools rely on various algorithmic measures like CAI, RSCU, Nc etc., and by using this information, both enhancement as well as reduction of protein expression is possible. On one hand, elucidation of the codon usage pattern is useful in optimizing protein expression. The information regarding enhanced protein expression will be useful in designing a subunit vaccine candidate, where a vaccine candidate is expressed in a prokaryotic/eukaryotic system. Apart from the subunit vaccine, codon optimization is also useful in constructing various other viral vectored vaccine candidates including vesicular stomatitis virus, rabies virus, canarypox virus, adeno-associated virus, measles virus, Newcastle disease virus and Venezuelan equine encephalitis virus harboring the G and N protein of NiV. On the other hand, contrary to protein expression enhancement, similar codon usage information can be used to reduce the NiV viral protein synthesis during replication of the pathogen. This rational systematic approach is called SAVE (synthetic attenuated vaccine engineering), by which choosing underrepresented codons and/or concomitant increase in rare dinucleotides like CpG and UpA, virus attenuation in a tunable manner is achieved. Codon usage information may also be obtained in a similar fashion for other viruses and investigations may be done for broad applicability.

## Author Contributions

RK, SS, UK, AA, RT, KD, JD, AM, and RS substantially contributed to the conception, design, and analysis of primary data, interpretation of data, checking and approving final version of the manuscript, and agreed to be accountable for its contents. RK and AM conceived the concept. SS, UK, and AA retrieved and analyzed the data and generated tables and figures under supervision of RK, JD, and RS. RK, AM, and KD wrote the manuscript. RT assisted in writing. KD, AM, and RS finalized the manuscript.

## Conflict of Interest Statement

The authors declare that the research was conducted in the absence of any commercial or financial relationships that could be construed as a potential conflict of interest.

## Footnotes

1 http://www.ncbi.nlm.nih.gov/GenBank

2 http://www.kazusa.or.jp/codon/

3 http://genomes.urv.es/CAIcal/

4 https://www.kazusa.or.jp/codon/

5 https://sourceforge.net/projects/codonw/files/codonw/Win32-Executables-1.4.2/

6 http://gtrnadb.ucsc.edu/
