# Supplementary material for: Analysis of Nipah Virus Codon Usage and Adaptation to Hosts
Source: Front Microbiol. 2019 May 8;10:886. doi: 10.3389/fmicb.2019.00886 (PMC6530375; doi:10.3389/fmicb.2019.00886)
Supplement: Supplementary file 1 [file Table_1.DOCX]

**Supplementary table 1:** Different coding sequences (G, F, M, N, L, P, C, V and W) used for codon bias study of Nipah virus (NiV) [Source: National Center for Biotechnology Information (NCBI); http://www.ncbi.nlm.nih.gov/GenBank].

| **S.**  **No.** | **Accession no.** | **Strain name** | **Gene/CDSs** | **Length (bp)** | **Species/ host** | **Isolated from** | **Year of isolation** |
| --- | --- | --- | --- | --- | --- | --- | --- |
| 1 | NC_002728.1 | -- | C/F/G/M/N/P/L/W/V | 18246 | -- | -- | - |
| 2 | JN808863.1 | NIVBGD2008RAJBARI | C/F/G/M/N/P/L/W/V | 18252 | *Homo sapiens* | throat swab | 2008 |
| 3 | JN808857.1 | NIVBGD2008MANIKGONJ | C/F/G/M/N/P/L/W/V | 18252 | *Homo sapiens* | throat swab | 2008 |
| 4 | FJ513078.1 | Ind-Nipah-07-FG | F/G/M/N/P/L | 18252 | *Homo sapiens* | lung tissue | 2007 |
| 5 | AY029768.1 | UMMC2 | C/F/G/M/N/P/L/V | 18246 | *Homo sapiens* | isolated from the throat secretion of an encephalitic patient | - |
| 6 | AF212302.2 | -- | C/F/G/M/N/P/L/V | 18246 | *--* | -- | - |
| 7 | AY988601.1 | -- | C/F/G/M/N/P/L/W/V | 18252 | *Homo sapiens* | -- | 2004 |
| 8 | AY029767.1 | UMMC1 | C/F/G/M/N/P/L/V | 18246 | *Homo sapiens* | CSF of an encephalitic patient | - |
| 9 | AJ564621.1 | NV/MY/99/VRI-2794 | F/G/M/N/P/L | 18246 | -- | -- | - |
| 10 | AJ627196.1 | NV/MY/99/VRI-0626 | F/G/M/N/P/L | 18246 | *Sus scrofa* (pig) | -- | - |
| 11 | JN808864.1 | NIVBGD2010FARIDPUR | C/F/G/M/N/P/L/W/V | 18167 | *Homo sapiens* | throat swab | 2010 |
| 12 | KY425655.1 | IRF0158 | C/F/M/N/P/L/W/V | 18214 | *Homo sapiens* | Vero C1008 (E6) cells | 1999 |
| 13 | AJ564623.1 | NV/MY/99/UM-0128 | G/M/N/P/L | 18246 | *--* | -- | - |
| 14 | AJ564622.1 | NV/MY/99/VRI-1413 | G/M/N/P/L | 18246 | -- | -- | - |
| 15 | AF376747.1 | -- | C/G/M/N/P/V | 11200 | *Pteropus hypomelanus* (Island flying-fox) | -- | - |
| 16 | KY425646.1 | IRF0160 | C/F/G/M/N/P/L/W/V | 18212 | *Homo sapiens* | Vero C1008 (E6) cells | 1999 |
| 17 | JF899342.1 | 1056660 | F | 552 | *Pteropus giganteus* (bat) | -- | 2010 |
| 18 | AF238466.1 | -- | F | 2337 | -- | -- | - |
| 19 | DI399829.1 | KR 1020140053906-A/3 | G | 1809 | -- | -- | - |
| 20 | HM545086.1 | -- | G | 1809 | *Pteropus vampyrus* | urine | 2008 |
| 21 | KY425655.1 | IRF0158 | G | 18214 | *Homo sapiens* | Vero C1008 (E6) cells | Mar-2015 |
| 22 | AF238467.1 | -- | G | 2546 | *--* | -- | - |
| 23 | AY858111.1 | NiV/KHM/CSUR381 | G | 1809 | *Pteropus lylei* | urine | - |
| 24 | JF899340.1 | 1056660 | G | 954 | *Pteropus giganteus* | -- | 2010 |
| 25 | KT163256.1 | 33654 | N | 1599 | *Pteropus lylei* | urine | 2011 |
| 26 | KT163252.1 | 3054 | N | 1599 | *Pteropus lylei* | urine | 2011 |
| 27 | KT163255.1 | 14653 | N | 1599 | *Pteropus lylei* | urine | 2010 |
| 28 | KT163254.1 | 15553 | N | 1599 | *Pteropus lylei* | urine | 2010 |
| 29 | KT163253.1 | 15053 | N | 1599 | *Pteropus lylei* | urine | 2010 |
| 30 | KT163251.1 | 1054 | N | 1599 | *Pteropus lylei* | urine | 2010 |
| 31 | KT163250.1 | 8554 | N | 1599 | *Pteropus hypomelanus* | urine | 2011 |
| 32 | KT163249.1 | 1353 | N | 1599 | *Pteropus hypomelanus* | urine | 2010 |
| 33 | KT163248.1 | 16454 | N | 1599 | *Pteropus hypomelanus* | urine | 2011 |
| 34 | KT163247.1 | 1753 | N | 1599 | *Pteropus hypomelanus* | urine | 2010 |
| 35 | KM034755.1 | NiV_KD_C313_KH13 | N | 1599 | *Pteropus lylei* | -- | 2013 |
| 36 | JN808862.1 | NIVBGD2004RAJSHAHI | N | 1599 | *Homo sapiens* | throat swab | 2004 |
| 37 | JN808861.1 | NIVBGD2004RAJBARI2 | N | 1599 | *Homo sapiens* | throat swab | 2004 |
| 38 | JN808860.1 | NIVBGD2010GOPALGANJ | N | 1599 | *Homo sapiens* | throat swab | 2010 |
| 39 | JN808859.1 | NIVBGD2010FARIDPUR2 | N | 1599 | *Homo sapiens* | throat swab | 2010 |
| 40 | JN808858.1 | NIVBGD2004FARIDPUR | N | 1599 | *Homo sapiens* | throat swab | 2004 |
| 41 | AY858110.1 | NiV/KHM/CSUR381 | N | 1599 | *Pteropus lylei* | urine | - |
| 42 | HM545087.1 | Malaysian phosphoprotein | P | 2130 | *Pteropus vampyrus* | urine | 2008 |
| 43 | HM545088.1 | Malaysian polymerase | L | 6735 | *Pteropus vampyrus* | urine | 2008 |
