# Supplementary material for: Analysis of Nipah Virus Codon Usage and Adaptation to Hosts
Source: Front Microbiol. 2019 May 8;10:886. doi: 10.3389/fmicb.2019.00886 (PMC6530375; doi:10.3389/fmicb.2019.00886)
Supplement: Supplementary file 2 [file Table_2.DOCX]

**Supplementary table 2:** Nucleotides composition of Nipah virus (NiV) genome at third codon position (U3%, G3%, C3% and A3%), overall AU%, AU3% and GC% composition along with GC12 (the average of GC1 and GC2) and GC3% analysis.

| **Coding Sequences (CDSs)/**  **Accession number (NiV)** | **Gene** | **Length** | **A%** | **C%** | **U%** | **G%** | **A3%** | **C3%** | **U3%** | **G3%** | **AU%** | **GC%** | **GC1** | **GC2** | **GC12** | **AU3%** | **GC3%** | **Nc** | **CAI** | **GRAVY** | **AROMO** |
| --- | --- | --- | --- | --- | --- | --- | --- | --- | --- | --- | --- | --- | --- | --- | --- | --- | --- | --- | --- | --- | --- |
| Coding sequence C (protein C encoded by gene P) | | | | | | | | | | | | | | | | | | | | | |
| NC002728.1:2428-2928 | C | 501 | 34.13 | 17.56 | 22.95 | 25.35 | 29.34 | 15.57 | 16.17 | 38.92 | 57.09 | 42.90 | 41.90 | 32.30 | 37.10 | 45.51 | 54.50 | 50.50 | 0.76 | 0.79 | 0.078 |
| JN808863.1:2428-2928 |  |  | 33.13 | 18.16 | 22.36 | 26.35 | 27.54 | 16.77 | 15.57 | 40.12 | 55.49 | 44.50 | 43.70 | 32.90 | 38.30 | 43.11 | 56.90 | 49.00 | 0.76 | 0.79 | 0.078 |
| JN808857.1:2428-2928 |  |  | 33.13 | 18.16 | 22.36 | 26.35 | 27.54 | 16.77 | 15.57 | 40.12 | 55.49 | 44.50 | 43.70 | 32.90 | 38.30 | 43.11 | 56.90 | 49.00 | 0.76 | 0.79 | 0.078 |
| AY029768.1:2428-2928 |  |  | 34.13 | 17.56 | 22.95 | 25.35 | 29.34 | 15.57 | 16.17 | 38.92 | 57.09 | 42.90 | 41.90 | 32.30 | 37.10 | 45.51 | 54.50 | 50.50 | 0.76 | 0.79 | 0.078 |
| AF212302.2:2428-2928 |  |  | 34.13 | 17.56 | 22.95 | 25.35 | 29.34 | 15.57 | 16.17 | 38.92 | 57.09 | 42.90 | 41.90 | 32.30 | 37.10 | 45.51 | 54.50 | 50.50 | 0.76 | 0.79 | 0.078 |
| AY988601.1:2428-2928 |  |  | 33.13 | 17.96 | 22.55 | 26.35 | 27.54 | 16.17 | 16.17 | 40.12 | 55.69 | 44.30 | 43.70 | 32.90 | 38.30 | 43.71 | 56.30 | 49.00 | 0.76 | 0.78 | 0.078 |
| AY029767.1:2428-2928 |  |  | 34.13 | 17.56 | 22.95 | 25.35 | 29.34 | 15.57 | 16.17 | 38.92 | 57.09 | 42.90 | 41.90 | 32.30 | 37.10 | 45.51 | 54.50 | 50.50 | 0.76 | 0.79 | 0.078 |
| JN808864.1:2373-2873 |  |  | 33.33 | 17.96 | 22.55 | 26.15 | 28.14 | 16.17 | 16.17 | 39.52 | 55.89 | 44.10 | 43.70 | 32.90 | 38.30 | 44.31 | 55.70 | 48.80 | 0.76 | 0.79 | 0.078 |
| AF376747.1:2373-2873 |  |  | 34.13 | 17.56 | 22.95 | 25.35 | 29.34 | 15.57 | 16.17 | 38.92 | 57.09 | 42.90 | 41.90 | 32.30 | 37.10 | 45.51 | 54.50 | 50.50 | 0.76 | 0.79 | 0.078 |
| KY425655.1:2412-2912 |  |  | 34.13 | 17.56 | 22.95 | 25.35 | 29.34 | 15.57 | 16.17 | 38.92 | 57.09 | 42.90 | 41.90 | 32.30 | 37.10 | 45.51 | 54.50 | 50.50 | 0.76 | 0.79 | 0.078 |
| KY425646.1:2415-2915 |  |  | 34.13 | 17.56 | 22.95 | 25.35 | 29.34 | 15.57 | 16.17 | 38.92 | 57.09 | 42.90 | 41.90 | 32.30 | 37.10 | 45.51 | 54.50 | 50.50 | 0.76 | 0.79 | 0.078 |
| Coding sequence F (encodes fusion protein) | | | | | | | | | | | | | | | | | | | | | |
| NC_002728.1:6654-8294 | F | 1641 | 33.27 | 18.10 | 28.58 | 20.05 | 30.90 | 19.20 | 31.99 | 17.92 | 61.85 | 38.10 | 41.30 | 36.00 | 38.65 | 62.89 | 37.10 | 49.50 | 0.71 | 0.77 | 0.070 |
| JN808863.1:6660-8300 |  |  | 33.03 | 18.40 | 28.46 | 20.11 | 29.80 | 19.93 | 31.63 | 18.65 | 61.49 | 38.50 | 41.00 | 36.00 | 38.50 | 61.43 | 38.60 | 51.70 | 0.70 | 0.78 | 0.071 |
| JN808857.1:6660-8300 |  |  | 33.03 | 18.40 | 28.46 | 20.11 | 29.80 | 19.93 | 31.63 | 18.65 | 61.49 | 38.50 | 41.00 | 36.00 | 38.50 | 61.43 | 38.60 | 51.70 | 0.70 | 0.78 | 0.071 |
| FJ513078.1:6660-8300 |  |  | 33.03 | 18.28 | 28.52 | 20.17 | 29.80 | 19.56 | 31.81 | 18.83 | 61.55 | 38.50 | 41.00 | 36.00 | 38.50 | 61.61 | 38.40 | 51.70 | 0.70 | 0.77 | 0.071 |
| AY029768.1:6654-8294 |  |  | 33.27 | 18.10 | 28.58 | 20.05 | 30.90 | 19.20 | 31.99 | 17.92 | 61.85 | 38.10 | 41.30 | 36.00 | 38.65 | 62.89 | 37.10 | 49.50 | 0.71 | 0.77 | 0.070 |
| AF212302.2:6654-8294 |  |  | 33.27 | 18.10 | 28.58 | 20.05 | 30.90 | 19.20 | 31.99 | 17.92 | 61.85 | 38.10 | 41.30 | 36.00 | 38.65 | 62.89 | 37.10 | 49.50 | 0.71 | 0.77 | 0.070 |
| AY988601.1:6660-8300 |  |  | 33.03 | 18.34 | 28.46 | 20.17 | 29.98 | 19.74 | 31.63 | 18.65 | 61.49 | 38.50 | 40.80 | 36.40 | 38.60 | 61.61 | 38.40 | 51.70 | 0.70 | 0.77 | 0.071 |
| AY029767.1:6654-8294 |  |  | 33.27 | 18.10 | 28.58 | 20.05 | 30.90 | 19.20 | 31.99 | 17.92 | 61.85 | 38.10 | 41.30 | 36.00 | 38.65 | 62.89 | 37.10 | 49.50 | 0.71 | 0.77 | 0.070 |
| AJ564621.1:6654-8294 |  |  | 33.27 | 18.04 | 28.64 | 20.05 | 30.90 | 19.01 | 32.18 | 17.92 | 61.91 | 38.10 | 41.30 | 36.00 | 38.65 | 63.07 | 36.90 | 49.40 | 0.71 | 0.77 | 0.070 |
| AJ627196.1:6654-8294 |  |  | 33.39 | 17.98 | 28.70 | 19.93 | 31.26 | 19.20 | 31.99 | 17.55 | 62.10 | 37.90 | 41.10 | 35.80 | 38.45 | 63.25 | 36.70 | 49.40 | 0.71 | 0.77 | 0.070 |
| JN808864.1:6605-8245 |  |  | 33.09 | 18.46 | 28.28 | 20.17 | 29.98 | 20.11 | 31.08 | 18.83 | 61.37 | 38.60 | 41.00 | 36.00 | 38.50 | 61.06 | 38.90 | 51.90 | 0.71 | 0.78 | 0.071 |
| KY425655.1:6638-8278 |  |  | 33.27 | 18.10 | 28.58 | 20.05 | 30.90 | 19.20 | 31.99 | 17.92 | 61.85 | 38.10 | 41.30 | 36.00 | 38.65 | 62.89 | 37.10 | 49.50 | 0.71 | 0.77 | 0.070 |
| AJ564623.1:6654-8294 |  |  | 33.27 | 18.10 | 28.58 | 20.05 | 30.90 | 19.20 | 31.99 | 17.92 | 61.85 | 38.10 | 41.30 | 36.00 | 38.65 | 62.89 | 37.10 | 49.50 | 0.71 | 0.77 | 0.070 |
| AJ564622.1:6654-8294 |  |  | 33.27 | 18.10 | 28.58 | 20.05 | 30.90 | 19.20 | 31.99 | 17.92 | 61.85 | 38.10 | 41.30 | 36.00 | 38.65 | 62.89 | 37.10 | 49.50 | 0.71 | 0.77 | 0.070 |
| KY425646.1:6641-8281 |  |  | 33.27 | 18.10 | 28.58 | 20.05 | 30.90 | 19.20 | 31.99 | 17.92 | 61.85 | 38.10 | 41.30 | 36.00 | 38.65 | 62.89 | 37.10 | 49.50 | 0.71 | 0.77 | 0.070 |
| AF238466.1 |  |  | 33.27 | 18.10 | 28.58 | 20.05 | 30.90 | 19.20 | 31.99 | 17.92 | 61.85 | 38.10 | 41.30 | 36.00 | 38.65 | 62.89 | 37.10 | 49.50 | 0.71 | 0.77 | 0.070 |
| JF899342.1 | F | 552 | 31.34 | 20.11 | 27.90 | 20.65 | 29.35 | 20.65 | 31.52 | 18.48 | 59.24 | 40.80 | 41.30 | 41.80 | 41.55 | 60.87 | 39.10 | 54.90 | 0.73 | 0.79 | 0.060 |
| Coding sequence G (encodes glycoprotein) | | | | | | | | | | | | | | | | | | | | | |
| NC_002728.1:8943-10751 | G | 1809 | 33.55 | 19.57 | 26.64 | 20.23 | 31.51 | 20.73 | 30.51 | 17.25 | 60.20 | 39.80 | 42.80 | 38.60 | 40.70 | 62.02 | 38.00 | 52.00 | 0.73 | 0.83 | 0.088 |
| JN808863.1:8949-10757 |  |  | 33.67 | 19.73 | 26.31 | 20.29 | 30.68 | 21.39 | 29.85 | 18.08 | 59.98 | 40.00 | 43.30 | 37.30 | 40.30 | 60.53 | 39.50 | 50.50 | 0.74 | 0.83 | 0.090 |
| JN808857.1:8949-10757 |  |  | 33.67 | 19.73 | 26.31 | 20.29 | 30.68 | 21.39 | 29.85 | 18.08 | 59.98 | 40.00 | 43.30 | 37.30 | 40.30 | 60.53 | 39.50 | 50.50 | 0.74 | 0.83 | 0.090 |
| FJ513078.1:8949-10757 |  |  | 33.61 | 19.79 | 26.26 | 20.34 | 30.68 | 21.56 | 29.68 | 18.08 | 59.87 | 40.10 | 43.40 | 37.30 | 40.35 | 60.36 | 39.60 | 50.30 | 0.74 | 0.84 | 0.090 |
| AY029768.1:8943-10751 |  |  | 33.55 | 19.57 | 26.64 | 20.23 | 31.51 | 20.73 | 30.51 | 17.25 | 60.20 | 39.80 | 42.80 | 38.60 | 40.70 | 62.02 | 38.00 | 52.00 | 0.73 | 0.83 | 0.088 |
| AF212302.2:8943-10751 |  |  | 33.55 | 19.57 | 26.64 | 20.23 | 31.51 | 20.73 | 30.51 | 17.25 | 60.20 | 39.80 | 42.80 | 38.60 | 40.70 | 62.02 | 38.00 | 52.00 | 0.73 | 0.83 | 0.088 |
| AY988601.1:8949-10757 |  |  | 33.78 | 19.79 | 26.20 | 20.23 | 31.01 | 21.56 | 29.52 | 17.91 | 59.98 | 40.00 | 43.30 | 37.30 | 40.30 | 60.53 | 39.50 | 49.90 | 0.74 | 0.84 | 0.090 |
| AY029767.1:8943-10751 |  |  | 33.55 | 19.57 | 26.64 | 20.23 | 31.51 | 20.73 | 30.51 | 17.25 | 60.20 | 39.80 | 42.80 | 38.60 | 40.70 | 62.02 | 38.00 | 52.00 | 0.73 | 0.83 | 0.088 |
| AJ564621.1:8943-10751 |  |  | 33.61 | 19.57 | 26.64 | 20.18 | 31.67 | 20.73 | 30.51 | 17.08 | 60.25 | 39.70 | 42.80 | 38.60 | 40.70 | 62.19 | 37.80 | 52.00 | 0.73 | 0.83 | 0.088 |
| AJ627196.1:8943-10751 |  |  | 33.55 | 19.57 | 26.59 | 20.29 | 31.51 | 20.73 | 30.51 | 17.25 | 60.14 | 39.90 | 43.00 | 38.60 | 40.80 | 62.02 | 38.00 | 52.10 | 0.73 | 0.83 | 0.088 |
| DI399829.1_KR  1020140053906-A/3 |  |  | 33.55 | 19.57 | 26.64 | 20.23 | 31.51 | 20.73 | 30.51 | 17.25 | 60.20 | 39.80 | 42.80 | 38.60 | 40.70 | 62.02 | 38.00 | 52.00 | 0.73 | 0.83 | 0.088 |
| JN808864.1:8894-10702 |  |  | 33.72 | 19.73 | 26.31 | 20.23 | 31.01 | 21.23 | 29.85 | 17.91 | 60.03 | 40.00 | 43.30 | 37.50 | 40.40 | 60.86 | 39.10 | 49.80 | 0.74 | 0.84 | 0.090 |
| HM545086.1 |  |  | 33.44 | 19.73 | 26.37 | 20.45 | 31.18 | 21.06 | 30.18 | 17.58 | 59.81 | 40.20 | 43.30 | 38.60 | 40.95 | 61.36 | 38.60 | 52.40 | 0.73 | 0.83 | 0.088 |
| AF376747.1:8888-10696 |  |  | 33.61 | 19.51 | 26.70 | 20.18 | 31.67 | 20.56 | 30.68 | 17.08 | 60.31 | 39.70 | 42.80 | 38.60 | 40.70 | 62.35 | 37.60 | 51.90 | 0.73 | 0.83 | 0.088 |
| KY425655.1:8927-10735 |  |  | 33.55 | 19.57 | 26.64 | 20.23 | 31.51 | 20.73 | 30.51 | 17.25 | 60.20 | 39.80 | 42.80 | 38.60 | 40.70 | 62.02 | 38.00 | 52.00 | 0.73 | 0.83 | 0.088 |
| AF238467.1:234-2042 |  |  | 33.55 | 19.57 | 26.64 | 20.23 | 31.51 | 20.73 | 30.51 | 17.25 | 60.20 | 39.80 | 42.80 | 38.60 | 40.70 | 62.02 | 38.00 | 52.00 | 0.73 | 0.83 | 0.088 |
| AJ564623.1:8943-10751 |  |  | 33.61 | 19.57 | 26.64 | 20.18 | 31.67 | 20.73 | 30.51 | 17.08 | 60.25 | 39.70 | 42.80 | 38.60 | 40.70 | 62.19 | 37.80 | 52.00 | 0.73 | 0.83 | 0.088 |
| AY858111.1 |  |  | 33.72 | 19.29 | 26.81 | 20.18 | 31.84 | 20.07 | 31.01 | 17.08 | 60.53 | 39.50 | 42.60 | 38.60 | 40.60 | 62.85 | 37.10 | 51.00 | 0.73 | 0.82 | 0.088 |
| AJ564622.1:8943-10751 |  |  | 33.55 | 19.57 | 26.64 | 20.23 | 31.51 | 20.73 | 30.51 | 17.25 | 60.20 | 39.80 | 42.80 | 38.60 | 40.70 | 62.02 | 38.00 | 52.00 | 0.73 | 0.83 | 0.088 |
| KY425646.1:8930-10738 |  |  | 33.55 | 19.57 | 26.64 | 20.23 | 31.51 | 20.73 | 30.51 | 17.25 | 60.20 | 39.80 | 42.80 | 38.60 | 40.70 | 62.02 | 38.00 | 52.00 | 0.73 | 0.83 | 0.088 |
| JF899340.1 | G | 954 | 31.34 | 19.71 | 28.30 | 20.65 | 30.19 | 22.33 | 33.02 | 14.47 | 59.64 | 40.40 | 45.60 | 38.70 | 42.15 | 63.21 | 36.80 | 50.10 | 0.72 | 0.78 | 0.126 |
| Coding sequence M (encodes matrix protein) | | | | | | | | | | | | | | | | | | | | | |
| NC_002728.1:5108-6166 | M | 1059 | 29.46 | 19.17 | 27.95 | 23.42 | 20.96 | 23.23 | 33.14 | 22.66 | 57.41 | 42.60 | 45.30 | 36.50 | 40.90 | 54.11 | 45.90 | 54.20 | 0.76 | 0.72 | 0.099 |
| JN808863.1:5108-6166 |  |  | 29.18 | 19.36 | 27.95 | 23.51 | 20.96 | 24.08 | 32.86 | 22.10 | 57.13 | 42.90 | 45.90 | 36.50 | 41.20 | 53.82 | 46.20 | 56.20 | 0.76 | 0.72 | 0.099 |
| JN808857.1:5108-6166 |  |  | 29.18 | 19.36 | 27.95 | 23.51 | 20.96 | 24.08 | 32.86 | 22.10 | 57.13 | 42.90 | 45.90 | 36.50 | 41.20 | 53.82 | 46.20 | 56.20 | 0.76 | 0.72 | 0.099 |
| FJ513078.1:5108-6166 |  |  | 29.18 | 19.36 | 28.05 | 23.42 | 20.96 | 24.08 | 33.14 | 21.81 | 57.22 | 42.80 | 45.90 | 36.50 | 41.20 | 54.11 | 45.90 | 56.10 | 0.76 | 0.72 | 0.099 |
| AY029768.1:5108-6166 |  |  | 29.46 | 19.17 | 27.95 | 23.42 | 20.96 | 23.23 | 33.14 | 22.66 | 57.41 | 42.60 | 45.30 | 36.50 | 40.90 | 54.11 | 45.90 | 54.20 | 0.76 | **0.72** | 0.099 |
| AF212302.2:5108-6166 |  |  | 29.46 | 19.17 | 27.95 | 23.42 | 20.96 | 23.23 | 33.14 | 22.66 | 57.41 | 42.60 | 45.30 | 36.50 | 40.90 | 54.11 | 45.90 | 54.20 | 0.76 | 0.72 | 0.099 |
| AY029767.1:5108-6166 |  |  | 29.46 | 19.17 | 27.95 | 23.42 | 20.96 | 23.23 | 33.14 | 22.66 | 57.41 | 42.60 | 45.30 | 36.50 | 40.90 | 54.11 | 45.90 | 54.20 | 0.76 | 0.72 | 0.099 |
| AJ564621.1:5108-6166 |  |  | 29.46 | 19.17 | 27.95 | 23.42 | 20.96 | 23.23 | 33.14 | 22.66 | 57.41 | 42.60 | 45.30 | 36.50 | 40.90 | 54.11 | 45.90 | 54.20 | 0.76 | 0.72 | 0.099 |
| AJ627196.1:5108-6166 |  |  | 29.27 | 19.26 | 27.86 | 23.61 | 20.68 | 23.51 | 32.86 | 22.95 | 57.13 | 42.90 | 45.60 | 36.50 | 41.05 | 53.54 | 46.50 | 54.00 | 0.77 | 0.72 | 0.099 |
| JN808864.1:5053-6111 |  |  | 29.18 | 19.36 | 27.95 | 23.51 | 20.96 | 24.08 | 32.86 | 22.10 | 57.13 | 42.90 | 45.90 | 36.50 | 41.20 | 53.82 | 46.20 | 56.30 | 0.76 | 0.72 | 0.099 |
| AF376747.1:5053-6111 |  |  | 29.46 | 19.17 | 27.95 | 23.42 | 20.96 | 23.23 | 33.14 | 22.66 | 57.41 | 42.60 | 45.30 | 36.50 | 40.90 | 54.11 | 45.90 | 54.20 | 0.76 | 0.72 | 0.099 |
| KY425655.1:5092-6150 |  |  | 29.46 | 19.17 | 27.95 | 23.42 | 20.96 | 23.23 | 33.14 | 22.66 | 57.41 | 42.60 | 45.30 | 36.50 | 40.90 | 54.11 | 45.90 | 54.20 | 0.76 | 0.72 | 0.099 |
| AJ564623.1:5108-6166 |  |  | 29.46 | 19.17 | 27.95 | 23.42 | 20.96 | 23.23 | 33.14 | 22.66 | 57.41 | 42.60 | 45.30 | 36.50 | 40.90 | 54.11 | 45.90 | 54.20 | 0.76 | 0.72 | 0.099 |
| AJ564622.1:5108-6166 |  |  | 29.46 | 19.17 | 27.95 | 23.42 | 20.96 | 23.23 | 33.14 | 22.66 | 57.41 | 42.60 | 45.30 | 36.50 | 40.90 | 54.11 | 45.90 | 54.20 | 0.76 | 0.72 | 0.099 |
| KY425646.1:5095-6153 |  |  | 29.46 | 19.17 | 27.95 | 23.42 | 20.96 | 23.23 | 33.14 | 22.66 | 57.41 | 42.60 | 45.30 | 36.50 | 40.90 | 54.11 | 45.90 | 54.20 | 0.76 | 0.72 | 0.099 |
| AY988601.1:2428-2928 | M | 501 | 33.13 | 17.96 | 22.55 | 26.35 | 27.54 | 16.17 | 16.17 | 40.12 | 55.69 | 44.30 | 43.70 | 32.90 | 38.30 | 43.71 | 56.30 | 49.00 | 0.76 | 0.78 | 0.078 |
| Coding sequence N (N protein responsible for encapsidating the viral genome) | | | | | | | | | | | | | | | | | | | | | |
| NC_002728.1:113-1711 | N | 1599 | 31.58 | 20.51 | 22.95 | 24.95 | 32.65 | 20.45 | 27.58 | 19.32 | 54.53 | 45.50 | 51.20 | 45.40 | 48.30 | 60.23 | 39.80 | 50.80 | 0.73 | 0.82 | 0.066 |
| JN808863.1:113-1711 |  |  | 31.02 | 20.08 | 23.51 | 25.39 | 30.58 | 19.14 | 29.27 | 21.01 | 54.53 | 45.50 | 51.40 | 44.80 | 48.10 | 59.85 | 40.20 | 51.30 | 0.72 | 0.79 | 0.066 |
| JN808857.1:113-1711 |  |  | 31.02 | 20.08 | 23.51 | 25.39 | 30.58 | 19.14 | 29.27 | 21.01 | 54.53 | 45.50 | 51.40 | 44.80 | 48.10 | 59.85 | 40.20 | 51.30 | 0.72 | 0.79 | 0.066 |
| FJ513078.1:113-1711 |  |  | 31.14 | 20.14 | 23.45 | 25.27 | 31.14 | 19.32 | 29.08 | 20.45 | 54.60 | 45.40 | 51.40 | 45.00 | 48.20 | 60.23 | 39.80 | 51.60 | 0.72 | 0.80 | 0.066 |
| AY029768.1:113-1711 |  |  | 31.58 | 20.51 | 22.95 | 24.95 | 32.65 | 20.45 | 27.58 | 19.32 | 54.53 | 45.50 | 51.20 | 45.40 | 48.30 | 60.23 | 39.80 | 50.80 | 0.73 | 0.82 | 0.066 |
| AF212302.2:113-1711 |  |  | 31.58 | 20.51 | 22.95 | 24.95 | 32.65 | 20.45 | 27.58 | 19.32 | 54.53 | 45.50 | 51.20 | 45.40 | 48.30 | 60.23 | 39.80 | 50.80 | 0.73 | 0.82 | 0.066 |
| AY988601.1:113-1711 |  |  | 31.21 | 20.14 | 23.58 | 25.08 | 31.14 | 19.32 | 29.46 | 20.08 | 54.78 | 45.20 | 51.40 | 44.80 | 48.10 | 60.60 | 39.40 | 51.00 | 0.72 | 0.80 | 0.066 |
| AY029767.1:113-1711 |  |  | 31.58 | 20.51 | 22.95 | 24.95 | 32.65 | 20.45 | 27.58 | 19.32 | 54.53 | 45.50 | 51.20 | 45.40 | 48.30 | 60.23 | 39.80 | 50.80 | 0.73 | 0.82 | 0.066 |
| AJ564621.1:113-1711 |  |  | 31.58 | 20.51 | 22.95 | 24.95 | 32.65 | 20.45 | 27.58 | 19.32 | 54.53 | 45.50 | 51.20 | 45.40 | 48.30 | 60.23 | 39.80 | 50.80 | 0.73 | 0.82 | 0.066 |
| AJ627196.1:113-1711 |  |  | 31.58 | 20.51 | 22.89 | 25.02 | 32.65 | 20.45 | 27.39 | 19.51 | 54.47 | 45.50 | 51.20 | 45.40 | 48.30 | 60.04 | 40.00 | 50.80 | 0.73 | 0.82 | 0.066 |
| JN808864.1:58-1656 |  |  | 31.27 | 20.08 | 23.58 | 25.08 | 31.14 | 19.14 | 29.46 | 20.26 | 54.85 | 45.20 | 51.40 | 44.70 | 48.05 | 60.60 | 39.40 | 51.10 | 0.72 | 0.80 | 0.066 |
| AF376747.1:58-1656 |  |  | 31.58 | 20.45 | 23.01 | 24.95 | 32.65 | 20.45 | 27.58 | 19.32 | 54.60 | 45.40 | 51.20 | 45.20 | 48.20 | 60.23 | 39.80 | 50.90 | 0.73 | 0.82 | 0.066 |
| KY425655.1:97-1695 |  |  | 31.58 | 20.51 | 22.95 | 24.95 | 32.65 | 20.45 | 27.58 | 19.32 | 54.53 | 45.50 | 51.20 | 45.40 | 48.30 | 60.23 | 39.80 | 50.80 | 0.73 | 0.82 | 0.066 |
| KT163256.1 |  |  | 31.14 | 20.26 | 23.45 | 25.14 | 31.14 | 19.89 | 28.89 | 20.08 | 54.60 | 45.40 | 51.60 | 44.70 | 48.15 | 60.04 | 40.00 | 51.40 | 0.72 | 0.80 | 0.066 |
| KT163252.1 |  |  | 31.27 | 20.26 | 23.39 | 25.08 | 31.33 | 19.70 | 28.89 | 20.08 | 54.66 | 45.30 | 51.40 | 44.80 | 48.10 | 60.23 | 39.80 | 51.30 | 0.72 | 0.81 | 0.066 |
| AJ564623.1:113-1711 |  |  | 31.58 | 20.51 | 22.95 | 24.95 | 32.65 | 20.45 | 27.58 | 19.32 | 54.53 | 45.50 | 51.20 | 45.40 | 48.30 | 60.23 | 39.80 | 50.80 | 0.73 | 0.82 | 0.066 |
| AJ564622.1:113-1711 |  |  | 31.58 | 20.51 | 22.95 | 24.95 | 32.65 | 20.45 | 27.58 | 19.32 | 54.53 | 45.50 | 51.20 | 45.40 | 48.30 | 60.23 | 39.80 | 50.80 | 0.73 | 0.82 | 0.066 |
| KY425646.1:100-1698 |  |  | 31.58 | 20.51 | 22.95 | 24.95 | 32.65 | 20.45 | 27.58 | 19.32 | 54.53 | 45.50 | 51.20 | 45.40 | 48.30 | 60.23 | 39.80 | 50.80 | 0.73 | 0.82 | 0.066 |
| KT163255.1 |  |  | 31.27 | 20.33 | 23.39 | 25.02 | 31.14 | 19.89 | 28.89 | 20.08 | 54.66 | 45.30 | 51.40 | 44.70 | 48.05 | 60.04 | 40.00 | 51.40 | 0.73 | 0.81 | 0.066 |
| KT163254.1 |  |  | 31.14 | 20.33 | 23.39 | 25.14 | 30.96 | 19.89 | 28.89 | 20.26 | 54.53 | 45.50 | 51.40 | 44.80 | 48.10 | 59.85 | 40.20 | 51.50 | 0.72 | 0.80 | 0.066 |
| KT163253.1 |  |  | 31.27 | 20.33 | 23.39 | 25.02 | 31.14 | 19.89 | 28.89 | 20.08 | 54.66 | 45.30 | 51.20 | 44.80 | 48.00 | 60.04 | 40.00 | 51.30 | 0.72 | 0.81 | 0.066 |
| KT163251.1 |  |  | 31.14 | 20.33 | 23.39 | 25.14 | 30.96 | 19.89 | 28.89 | 20.26 | 54.53 | 45.50 | 51.40 | 44.80 | 48.10 | 59.85 | 40.20 | 51.40 | 0.73 | 0.80 | 0.066 |
| KT163250.1 |  |  | 31.39 | 20.39 | 23.08 | 25.14 | 32.46 | 20.26 | 27.77 | 19.51 | 54.47 | 45.50 | 51.40 | 45.40 | 48.40 | 60.23 | 39.80 | 50.70 | 0.73 | 0.81 | 0.066 |
| KT163249.1 |  |  | 31.39 | 20.39 | 23.08 | 25.14 | 32.46 | 20.26 | 27.77 | 19.51 | 54.47 | 45.50 | 51.40 | 45.40 | 48.40 | 60.23 | 39.80 | 50.70 | 0.73 | 0.81 | 0.066 |
| KT163248.1 |  |  | 31.33 | 20.39 | 23.08 | 25.20 | 32.46 | 20.26 | 27.77 | 19.51 | 54.41 | 45.60 | 51.60 | 45.40 | 48.50 | 60.23 | 39.80 | 50.70 | 0.73 | 0.81 | 0.066 |
| KT163247.1 |  |  | 31.46 | 20.39 | 23.08 | 25.08 | 32.65 | 20.08 | 27.95 | 19.32 | 54.53 | 45.50 | 51.80 | 45.20 | 48.50 | 60.60 | 39.40 | 50.10 | 0.73 | 0.81 | 0.066 |
| KM034755.1 |  |  | 31.33 | 20.20 | 23.20 | 25.27 | 32.08 | 19.51 | 28.33 | 20.08 | 54.53 | 45.50 | 51.60 | 45.20 | 48.40 | 60.41 | 39.60 | 50.70 | 0.73 | 0.81 | 0.066 |
| JN808862.1 |  |  | 31.08 | 20.08 | 23.58 | 25.27 | 30.96 | 19.14 | 29.27 | 20.64 | 54.66 | 45.30 | 51.40 | 44.80 | 48.10 | 60.23 | 39.80 | 51.10 | 0.72 | 0.80 | 0.066 |
| JN808861.1 |  |  | 31.14 | 20.20 | 23.51 | 25.14 | 30.96 | 19.51 | 29.27 | 20.26 | 54.66 | 45.30 | 51.40 | 44.80 | 48.10 | 60.23 | 39.80 | 51.20 | 0.73 | 0.80 | 0.066 |
| JN808860.1 |  |  | 31.21 | 20.14 | 23.51 | 25.14 | 31.14 | 19.32 | 29.27 | 20.26 | 54.72 | 45.30 | 51.40 | 44.80 | 48.10 | 60.41 | 39.60 | 51.30 | 0.72 | 0.80 | 0.066 |
| JN808859.1 |  |  | 31.02 | 20.01 | 23.58 | 25.39 | 30.77 | 18.95 | 29.46 | 20.83 | 54.60 | 45.40 | 51.60 | 44.80 | 48.20 | 60.23 | 39.80 | 51.30 | 0.72 | 0.79 | 0.066 |
| JN808858.1 |  |  | 30.89 | 20.14 | 23.70 | 25.27 | 30.21 | 19.32 | 29.83 | 20.64 | 54.60 | 45.40 | 51.40 | 44.80 | 48.10 | 60.04 | 40.00 | 51.00 | 0.72 | 0.79 | 0.066 |
| AY858110.1 |  |  | 31.33 | 20.26 | 23.26 | 25.14 | 32.08 | 19.32 | 28.89 | 19.70 | 54.60 | 45.40 | 51.40 | 45.80 | 48.60 | 60.98 | 39.00 | 50.00 | 0.73 | 0.81 | 0.066 |
| Coding sequence P (encodes P protein) | | | | | | | | | | | | | | | | | | | | | |
| NC_002728.1:2406-4535 | P | 2130 | 33.85 | 19.81 | 22.86 | 23.47 | 31.27 | 18.73 | 30.42 | 19.58 | 56.71 | 43.30 | 53.90 | 37.60 | 45.75 | 61.69 | 38.30 | 53.10 | 0.74 | 0.85 | 0.049 |
| JN808863.1:2406-4535 |  |  | 33.52 | 19.62 | 22.96 | 23.90 | 31.41 | 18.17 | 30.85 | 19.58 | 56.48 | 43.50 | 54.40 | 38.50 | 46.45 | 62.25 | 37.70 | 51.90 | 0.75 | 0.84 | 0.049 |
| JN808857.1:2406-4535 |  |  | 33.52 | 19.62 | 22.96 | 23.90 | 31.41 | 18.17 | 30.85 | 19.58 | 56.48 | 43.50 | 54.40 | 38.50 | 46.45 | 62.25 | 37.70 | 51.90 | 0.75 | 0.84 | 0.049 |
| FJ513078.1:2406-4535 |  |  | 33.66 | 19.62 | 23.00 | 23.71 | 31.41 | 18.45 | 30.70 | 19.44 | 56.67 | 43.30 | 53.80 | 38.30 | 46.05 | 62.11 | 37.90 | 51.60 | 0.75 | 0.84 | 0.049 |
| AY029768.1:2406-4535 |  |  | 33.85 | 19.81 | 22.86 | 23.47 | 31.27 | 18.73 | 30.42 | 19.58 | 56.71 | 43.30 | 53.90 | 37.60 | 45.75 | 61.69 | 38.30 | 53.10 | 0.74 | 0.85 | 0.049 |
| AF212302.2:2406-4535 |  |  | 33.85 | 19.81 | 22.86 | 23.47 | 31.27 | 18.73 | 30.42 | 19.58 | 56.71 | 43.30 | 53.90 | 37.60 | 45.75 | 61.69 | 38.30 | 53.10 | 0.74 | 0.85 | 0.049 |
| AY988601.1:2406-4535 |  |  | 33.62 | 19.72 | 22.86 | 23.80 | 31.55 | 18.73 | 30.28 | 19.44 | 56.48 | 43.50 | 54.10 | 38.30 | 46.20 | 61.83 | 38.20 | 51.50 | 0.75 | 0.84 | 0.049 |
| AY029767.1:2406-4535 |  |  | 33.85 | 19.81 | 22.86 | 23.47 | 31.27 | 18.73 | 30.42 | 19.58 | 56.71 | 43.30 | 53.90 | 37.60 | 45.75 | 61.69 | 38.30 | 53.10 | 0.74 | 0.85 | 0.049 |
| AJ564621.1:2406-4535 |  |  | 33.85 | 19.81 | 22.86 | 23.47 | 31.27 | 18.73 | 30.42 | 19.58 | 56.71 | 43.30 | 53.90 | 37.60 | 45.75 | 61.69 | 38.30 | 53.10 | 0.74 | 0.85 | 0.049 |
| AJ627196.1:2406-4535 |  |  | 33.80 | 19.86 | 22.86 | 23.47 | 31.27 | 18.73 | 30.42 | 19.58 | 56.67 | 43.30 | 54.10 | 37.60 | 45.85 | 61.69 | 38.30 | 53.20 | 0.74 | 0.85 | 0.049 |
| JN808864.1:2351-4480 |  |  | 33.62 | 19.86 | 22.72 | 23.80 | 31.55 | 19.15 | 29.86 | 19.44 | 56.34 | 43.70 | 54.10 | 38.30 | 46.20 | 61.41 | 38.60 | 51.80 | 0.75 | 0.85 | 0.049 |
| AF376747.1:2351-4480 |  |  | 33.85 | 19.77 | 22.91 | 23.47 | 31.27 | 18.73 | 30.42 | 19.58 | 56.76 | 43.20 | 53.90 | 37.50 | 45.70 | 61.69 | 38.30 | 53.10 | 0.74 | 0.85 | 0.049 |
| KY425655.1:2390-4519 |  |  | 33.85 | 19.81 | 22.86 | 23.47 | 31.27 | 18.73 | 30.42 | 19.58 | 56.71 | 43.30 | 53.90 | 37.60 | 45.75 | 61.69 | 38.30 | 53.10 | 0.74 | 0.85 | 0.049 |
| AJ564623.1:2406-4535 |  |  | 33.85 | 19.81 | 22.86 | 23.47 | 31.27 | 18.73 | 30.42 | 19.58 | 56.71 | 43.30 | 53.90 | 37.60 | 45.75 | 61.69 | 38.30 | 53.10 | 0.74 | 0.85 | 0.049 |
| AJ564622.1:2406-4535 |  |  | 33.85 | 19.81 | 22.86 | 23.47 | 31.27 | 18.73 | 30.42 | 19.58 | 56.71 | 43.30 | 53.90 | 37.60 | 45.75 | 61.69 | 38.30 | 53.10 | 0.74 | 0.85 | 0.049 |
| KY425646.1:2393-4522 |  |  | 33.85 | 19.81 | 22.86 | 23.47 | 31.27 | 18.73 | 30.42 | 19.58 | 56.71 | 43.30 | 53.90 | 37.60 | 45.75 | 61.69 | 38.30 | 53.10 | 0.74 | 0.85 | 0.049 |
| HM545087.1 |  |  | 33.90 | 20.38 | 22.35 | 23.38 | 31.69 | 19.30 | 29.44 | 19.58 | 56.24 | 43.80 | 53.80 | 38.60 | 46.20 | 61.13 | 38.90 | 53.10 | 0.75 | 0.87 | 0.047 |
| Coding sequence L (encodes L protein, which possesses RNA polymerase activity) | | | | | | | | | | | | | | | | | | | | | |
| NC_002728.1:11412-18146 | L | 6735 | 33.91 | 17.79 | 28.54 | 19.76 | 31.76 | 16.79 | 32.96 | 18.49 | 62.45 | 37.60 | 42.90 | 34.50 | 38.70 | 64.72 | 35.30 | 49.60 | 0.72 | 0.78 | 0.090 |
| JN808863.1:11418-18152 |  |  | 33.69 | 18.08 | 28.36 | 19.87 | 30.91 | 17.91 | 32.20 | 18.98 | 62.05 | 38.00 | 42.60 | 34.30 | 38.45 | 63.12 | 36.90 | 50.60 | 0.73 | 0.78 | 0.090 |
| JN808857.1:11418-18152 |  |  | 33.69 | 18.08 | 28.36 | 19.87 | 30.91 | 17.91 | 32.20 | 18.98 | 62.05 | 38.00 | 42.60 | 34.30 | 38.45 | 63.12 | 36.90 | 50.60 | 0.73 | 0.78 | 0.090 |
| FJ513078.1:11418-18152 |  |  | 33.66 | 18.16 | 28.34 | 19.84 | 30.87 | 18.13 | 32.16 | 18.84 | 62.00 | 38.00 | 42.70 | 34.30 | 38.50 | 63.03 | 37.00 | 50.70 | 0.73 | 0.78 | 0.090 |
| AY029768.1:11412-18146 |  |  | 33.91 | 17.77 | 28.55 | 19.76 | 31.76 | 16.75 | 33.01 | 18.49 | 62.46 | 37.50 | 42.90 | 34.50 | 38.70 | 64.77 | 35.20 | 49.60 | 0.72 | 0.78 | 0.090 |
| AF212302.2:11412-18146 |  |  | 33.91 | 17.79 | 28.54 | 19.76 | 31.76 | 16.79 | 32.96 | 18.49 | 62.45 | 37.60 | 42.90 | 34.50 | 38.70 | 64.72 | 35.30 | 49.60 | 0.72 | 0.78 | 0.090 |
| AY988601.1:11418-18152 |  |  | 33.62 | 18.14 | 28.33 | 19.91 | 30.91 | 18.04 | 32.16 | 18.89 | 61.95 | 38.10 | 42.80 | 34.40 | 38.60 | 63.07 | 36.90 | 50.70 | 0.73 | 0.78 | 0.090 |
| AY029767.1:11412-18146 |  |  | 33.91 | 17.77 | 28.55 | 19.76 | 31.76 | 16.79 | 32.96 | 18.49 | 62.46 | 37.50 | 42.90 | 34.40 | 38.65 | 64.72 | 35.30 | 49.60 | 0.72 | 0.78 | 0.090 |
| AJ564621.1:11412-18146 |  |  | 33.91 | 17.77 | 28.55 | 19.76 | 31.76 | 16.79 | 32.96 | 18.49 | 62.46 | 37.50 | 42.90 | 34.40 | 38.65 | 64.72 | 35.30 | 49.60 | 0.72 | 0.78 | 0.090 |
| AJ627196.1:11412-18146 |  |  | 33.94 | 17.73 | 28.57 | 19.76 | 31.80 | 16.75 | 33.01 | 18.44 | 62.51 | 37.50 | 42.90 | 34.40 | 38.65 | 64.81 | 35.20 | 49.50 | 0.72 | 0.78 | 0.090 |
| JN808864.1:11363-18097 |  |  | 33.65 | 18.14 | 28.31 | 19.90 | 30.87 | 18.04 | 32.16 | 18.93 | 61.96 | 38.00 | 42.80 | 34.40 | 38.60 | 63.03 | 37.00 | 50.70 | 0.73 | 0.78 | 0.090 |
| KY425655.1:11396-18130 |  |  | 33.91 | 17.79 | 28.54 | 19.76 | 31.76 | 16.79 | 32.96 | 18.49 | 62.45 | 37.60 | 42.90 | 34.50 | 38.70 | 64.72 | 35.30 | 49.60 | 0.72 | 0.78 | 0.090 |
| AJ564623.1:11412-18146 |  |  | 33.91 | 17.76 | 28.57 | 19.76 | 31.76 | 16.79 | 32.96 | 18.49 | 62.48 | 37.50 | 42.90 | 34.40 | 38.65 | 64.72 | 35.30 | 49.60 | 0.72 | 0.78 | 0.090 |
| AJ564622.1:11412-18146 |  |  | 33.91 | 17.77 | 28.55 | 19.76 | 31.76 | 16.79 | 32.96 | 18.49 | 62.46 | 37.50 | 42.90 | 34.50 | 38.70 | 64.72 | 35.30 | 49.60 | 0.72 | 0.78 | 0.090 |
| KY425646.1:11399-18133 |  |  | 33.91 | 17.79 | 28.54 | 19.76 | 31.76 | 16.79 | 32.96 | 18.49 | 62.45 | 37.60 | 42.90 | 34.50 | 38.70 | 64.72 | 35.30 | 49.60 | 0.72 | 0.78 | 0.090 |
| HM545088.1 |  |  | 33.94 | 17.73 | 28.57 | 19.76 | 31.80 | 16.75 | 33.01 | 18.44 | 62.51 | 37.50 | 42.90 | 34.40 | 38.65 | 64.81 | 35.20 | 49.50 | 0.72 | 0.78 | 0.090 |
| Coding sequence W (Protein W encoded by gene P) | | | | | | | | | | | | | | | | | | | | | |
| NC_002728.1:2406-3623,3625-3757 | W | 1218 | 33.99 | 20.28 | 21.51 | 24.22 | 34.24 | 18.23 | 29.31 | 18.23 | 55.50 | 44.50 | 55.90 | 41.10 | 48.50 | 63.55 | 36.50 | 52.80 | 0.75 | 0.87 | 0.049 |
| JN808863.1:2406-3756 |  |  | 33.83 | 20.11 | 21.51 | 24.55 | 34.73 | 17.24 | 29.80 | 18.23 | 55.34 | 44.70 | 56.40 | 42.10 | 49.25 | 64.53 | 35.50 | 53.20 | 0.75 | 0.86 | 0.049 |
| JN808857.1:2406-3756 |  |  | 33.83 | 20.11 | 21.51 | 24.55 | 34.73 | 17.24 | 29.80 | 18.23 | 55.34 | 44.70 | 56.40 | 42.10 | 49.25 | 64.53 | 35.50 | 53.20 | 0.75 | 0.86 | 0.049 |
| AY988601.1:2406-3756 |  |  | 33.83 | 20.11 | 21.51 | 24.55 | 34.48 | 17.73 | 29.31 | 18.47 | 55.34 | 44.70 | 55.90 | 41.90 | 48.90 | 63.79 | 36.20 | 52.00 | 0.75 | 0.86 | 0.049 |
| JN808864.1:2351-3701 |  |  | 33.74 | 20.20 | 21.43 | 24.63 | 33.99 | 17.98 | 29.06 | 18.97 | 55.17 | 44.80 | 55.70 | 41.90 | 48.80 | 63.05 | 36.90 | 52.30 | 0.75 | 0.86 | 0.049 |
| KY425655.1:2390-3607,3609-3740 |  |  | 33.99 | 20.28 | 21.51 | 24.22 | 34.24 | 18.23 | 29.31 | 18.23 | 55.50 | 44.50 | 55.90 | 41.10 | 48.50 | 63.55 | 36.50 | 52.80 | 0.75 | 0.87 | 0.049 |
| KY425646.1:2393-3610,3612-3743 |  |  | 33.99 | 20.28 | 21.51 | 24.22 | 34.24 | 18.23 | 29.31 | 18.23 | 55.50 | 44.50 | 55.90 | 41.10 | 48.50 | 63.55 | 36.50 | 52.80 | 0.75 | 0.87 | 0.049 |
| Coding sequence V (Protein V encoded by gene P) | | | | | | | | | | | | | | | | | | | | | |
| NC_002728.1:2406-3624,3624-3775 | V | 1218 | 33.99 | 20.28 | 21.51 | 24.22 | 34.24 | 18.23 | 29.31 | 18.23 | 55.50 | 44.50 | 55.90 | 41.10 | 48.50 | 63.55 | 36.50 | 52.80 | 0.75 | 0.87 | 0.049 |
| JN808863.1:2406-3784 |  |  | 33.83 | 20.11 | 21.51 | 24.55 | 34.73 | 17.24 | 29.80 | 18.23 | 55.34 | 44.70 | 56.40 | 42.10 | 49.25 | 64.53 | 35.50 | 53.20 | 0.75 | 0.86 | 0.049 |
| JN808857.1:2406-3784 |  |  | 33.83 | 20.11 | 21.51 | 24.55 | 34.73 | 17.24 | 29.80 | 18.23 | 55.34 | 44.70 | 56.40 | 42.10 | 49.25 | 64.53 | 35.50 | 53.20 | 0.75 | 0.86 | 0.049 |
| AY029768.1:2406-3623,3623-3775 |  |  | 33.99 | 20.28 | 21.51 | 24.22 | 34.24 | 18.23 | 29.31 | 18.23 | 55.50 | 44.50 | 55.90 | 41.10 | 48.50 | 63.55 | 36.50 | 52.80 | 0.75 | 0.87 | 0.049 |
| AF212302.2:2406-3624,3624-3775 |  |  | 33.99 | 20.28 | 21.51 | 24.22 | 34.24 | 18.23 | 29.31 | 18.23 | 55.50 | 44.50 | 55.90 | 41.10 | 48.50 | 63.55 | 36.50 | 52.80 | 0.75 | 0.87 | 0.049 |
| AY988601.1:2406-3784 |  |  | 33.83 | 20.11 | 21.51 | 24.55 | 34.48 | 17.73 | 29.31 | 18.47 | 55.34 | 44.70 | 55.90 | 41.90 | 48.90 | 63.79 | 36.20 | 52.00 | 0.75 | 0.86 | 0.049 |
| AY029767.1:2406-3623,3623-3775 |  |  | 33.99 | 20.28 | 21.51 | 24.22 | 34.24 | 18.23 | 29.31 | 18.23 | 55.50 | 44.50 | 55.90 | 41.10 | 48.50 | 63.55 | 36.50 | 52.80 | 0.75 | 0.87 | 0.049 |
| JN808864.1:2351-3729 |  |  | 33.74 | 20.20 | 21.43 | 24.63 | 33.99 | 17.98 | 29.06 | 18.97 | 55.17 | 44.80 | 55.70 | 41.90 | 48.80 | 63.05 | 36.90 | 52.30 | 0.75 | 0.86 | 0.049 |
| AF376747.1:2351-3568,3568 |  |  | 33.99 | 20.20 | 21.59 | 24.22 | 34.24 | 18.23 | 29.31 | 18.23 | 55.58 | 44.40 | 55.90 | 40.90 | 48.40 | 63.55 | 36.50 | 52.90 | 0.75 | 0.87 | 0.049 |
| KY425655.1:2390-3608,3608-3759 |  |  | 33.99 | 20.28 | 21.51 | 24.22 | 34.24 | 18.23 | 29.31 | 18.23 | 55.50 | 44.50 | 55.90 | 41.10 | 48.50 | 63.55 | 36.50 | 52.80 | 0.75 | 0.87 | 0.049 |
| KY425646.1:2393-3611,3611-3762 |  |  | 33.99 | 20.28 | 21.51 | 24.22 | 34.24 | 18.23 | 29.31 | 18.23 | 55.50 | 44.50 | 55.90 | 41.10 | 48.50 | 63.55 | 36.50 | 52.80 | 0.75 | 0.87 | 0.049 |
| **Average Value**  **±SD** | | | **32.69**  **±1.51** | **19.34**  **±0.97** | **25.06**  **±2.67** | **22.90**  **±2.25** | **30.46**  **±3.50** | **19.36**  **±2.03** | **29.38**  **±4.29** | **20.80**  **±5.71** | **57.76**  **±2.84** | **42.24**  **±2.85** | **47.68**  **±5.39** | **38.86**  **±4.08** | **43.27**  **±4.34** | **59.84**  **±5.29** | **40.17**  **±5.29** | **51.57**  **±1.64** | **0.74**  **±0.02** | **0.80**  **±0.04** | **0.07**  **±0.02** |
