# Supplementary material for: Analysis of Nipah Virus Codon Usage and Adaptation to Hosts
Source: Front Microbiol. 2019 May 8;10:886. doi: 10.3389/fmicb.2019.00886 (PMC6530375; doi:10.3389/fmicb.2019.00886)
Supplement: Supplementary file 3 [file Table_3.DOCX]

**Supplementary table 3:** The percent occurrence of the dinucleotide ratio in the Nipah Virus (NiV) genome; its observed frequency as well as expected frequency (Odds ratio has been calculated by dividing observed values with the expected values of dinucleotides).

| **S. No.** | **Dinucleotide** | **% occurrence** | **Observed frequency** | **Expected frequency** | **Odds Ratio** |
| --- | --- | --- | --- | --- | --- |
| 1 | ApA | 10.4 | 0.109 | 0.063 | **1.747** |
| 2 | ApC | 5.5 | 0.056 | 0.063 | 0.888 |
| 3 | ApG | 8.1 | 0.074 | 0.063 | 1.183 |
| 4 | ApU | 9.1 | 0.099 | 0.063 | 1.585 |
| 5 | CpA | 7.7 | 0.075 | 0.063 | 1.195 |
| 6 | CpC | 3.8 | 0.035 | 0.063 | 0.553 |
| 7 | CpG | 1.8 | 0.016 | 0.063 | **0.258** |
| 8 | CpU | 5.7 | 0.057 | 0.063 | 0.917 |
| 9 | GpA | 8.8 | 0.080 | 0.063 | 1.277 |
| 10 | GpC | 3.6 | 0.031 | 0.063 | 0.495 |
| 11 | GpG | 5.0 | 0.044 | 0.063 | 0.702 |
| 12 | GpU | 4.5 | 0.045 | 0.063 | 0.725 |
| 13 | UpA | 6.3 | 0.074 | 0.063 | 1.183 |
| 14 | UpC | 6.0 | 0.062 | 0.063 | 0.988 |
| 15 | UpG | 7.0 | 0.066 | 0.063 | 1.056 |
| 16 | UpU | 6.7 | 0.078 | 0.063 | 1.247 |
